# Polysubstituted ferrocenes as tunable redox mediators

**DOI:** 10.3762/bjoc.14.86

**Published:** 2018-05-07

**Authors:** Sven D Waniek, Jan Klett, Christoph Förster, Katja Heinze

**Affiliations:** 1 Institute of Inorganic Chemistry and Analytical Chemistry, Johannes Gutenberg University Mainz, Duesbergweg 10–14, D-55128 Mainz, Germany

**Keywords:** cyclic voltammetry, ferrocene, paramagnetic NMR spectroscopy, redox mediator, spectroelectrochemistry

## Abstract

A series of four ferrocenyl ester compounds, 1-methoxycarbonyl- (**1**), 1,1’-bis(methoxycarbonyl)- (**2**), 1,1’,3-tris(methoxycarbonyl)- (**3**) and 1,1’,3,3’-tetrakis(methoxycarbonyl)ferrocene (**4**), has been studied with respect to their potential use as redox mediators. The impact of the number and position of ester groups present in **1**–**4** on the electrochemical potential *E*_1/2_ is correlated with the sum of Hammett constants. The **1**/**1^+^**–**4**/**4^+^** redox couples are chemically stable under the conditions of electrolysis as demonstrated by IR and UV–vis spectroelectrochemical methods. The energies of the C=O stretching vibrations of the ester moieties and the energies of the UV–vis absorptions of **1**–**4** and **1^+^**–**4^+^** correlate with the number of ester groups. Paramagnetic ^1^H NMR redox titration experiments give access to the chemical shifts of **1^+^**–**4^+^** and underline the fast electron self-exchange of the ferrocene/ferrocenium redox couples, required for rapid redox mediation in organic electrosynthesis.

## Introduction

Since its discovery, ferrocene (FcH) has been established as versatile redox-active building block [1–3]. Ferrocene can be reversibly oxidized to the 17 valence electron ferrocenium cation (FcH^+^) at a useful electrochemical potential (FcH/FcH^+^ +630 mV vs NHE; +380 mV vs SCE in CH_3_CN) [4]. The 0/+ redox couple of ferrocene and its derivatives possesses high electron self-exchange rates *k*_ex_ = 10^6^–10^7^ M^−1^ s^−1^, remarkably independent on the electrolyte and solvent [5–6]. Both, the ferrocene/ferrocenium and the decamethylferrocene/decamethylferrocenium redox couples are well established as internal reference redox systems for electrochemical analyses in non-aqueous media [7–10]. Important requirements for redox couples with respect to useful applications are: (i) Both components of the redox couple should be soluble. (ii) Homogeneous and heterogeneous electron-transfer (ET) reactions should be fast. (iii) Both components should be stable under the electrolysis conditions and should not react irreversibly with any component of the supporting electrolyte [8]. In general, the redox mediators used as redox catalysts in indirect organic electrosyntheses should comprise the same characteristics [11–14]. A mediator is a reversible redox couple with a fast ET between itself and the electrode (heterogeneous) and between itself and the substrate (homogeneous). The benefit of the presence of a mediator is the switch of the sluggish heterogeneous electron transfer between electrode and substrate to a rapid homogeneous redox reaction between mediator and substrate. Further, the mediator’s redox potential must be below or above of that of the substrate for oxidation or reduction processes, respectively. This avoids the often kinetically hindered direct ET between electrode and substrate and diminishes overoxidation or overreduction of the substrate.

**Scheme 1:**
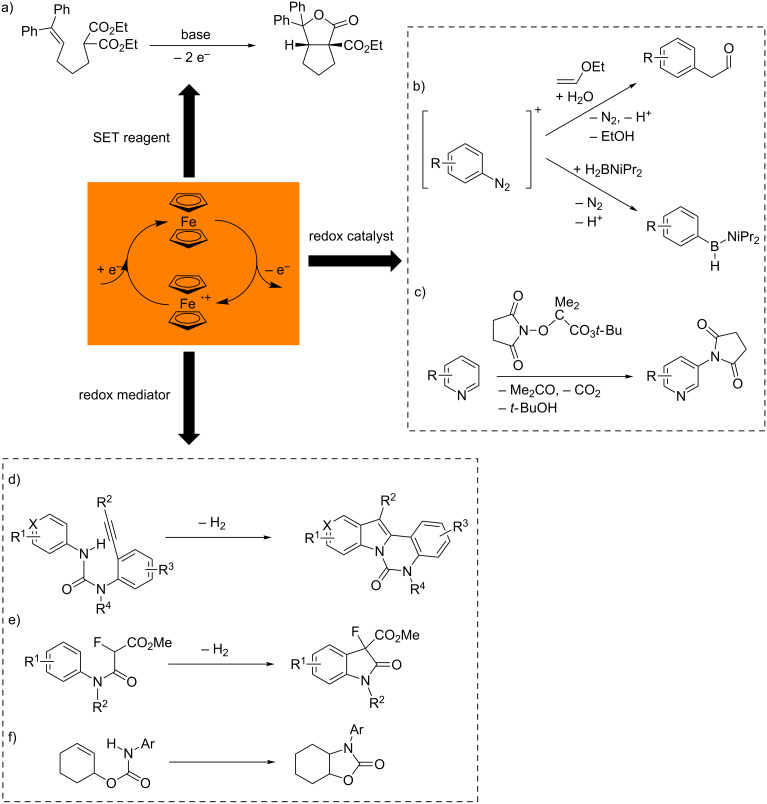
Selected transformations with ferrocene/ferrocenium as SET reagents (a) [27], catalyzed (b,c) [29–31] and mediated transformations (d–f) [34–36] by the ferrocene/ferrocenium redox couple.

Redox-active ferrocenyl derivatives find application in redox flow batteries [15], with water soluble (ferrocenylmethyl)ammonium salts acting as catholytes. Ferrocene dicarboxylic acid has been described as mediator for the voltammetric determination of glutathione in hemolized erythrocytes [16]. (Substituted) ferrocenium salts were successfully employed as single-electron transfer (SET) reagents in organic syntheses [17–28]. Tuning of the electrochemical potential of substituted ferrocenium salts promoted a selective oxidative bicyclization reaction under mild conditions (Scheme 1a) [27]. Ferrocene and decamethylferrocene act as redox catalysts in Meerwein arylation reactions [29], borylations of arenediazonium salts [30] and in C–H imidation reactions of (hetero)arenes [31] (Scheme 1b,c). Ferrocene has been used as redox mediator for the electrochemical modification of carbon surfaces via electrochemical oxidation of carboxylates [32–33], as mediator for dehydrogenative coupling reactions [34–35] and for olefin hydroamidations [36] (Scheme 1d–f).

**Scheme 2:**
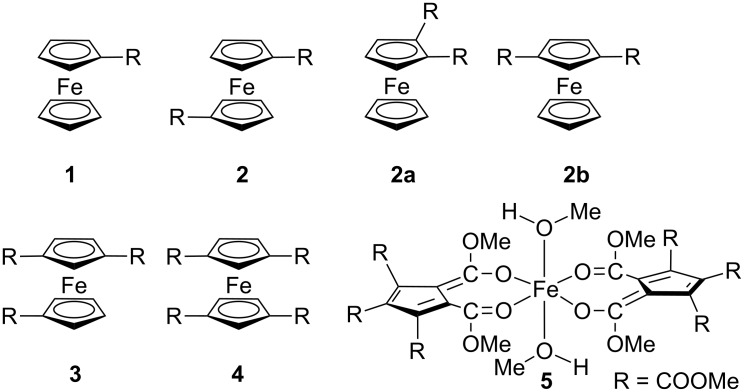
Methyl esters of ferrocene carboxylic acids **1** [45–46], **2** [47–49], **2a** [50], **2b** [51–52], **3**, **4** [54] and pseudo octahedral high-spin iron(II) complex **5** with pentakis(methoxycarbonyl)cyclopentadienyl ligands [55–56].

For potential applications of ferrocene derivatives as redox mediators or SET reagents, it is crucial to adjust the electrochemical potential to the potential of the substrate. The electrochemical potential of the ferrocene/ferrocenium redox couple strongly depends on the number and types of substituents [27,37–44]. One major drawback of changing the substituents is the dramatic change in chemical reactivity of ferrocene derivatives, e.g., ligand substitutions, apart from the solely intended tuning of the redox potential. A single class of ferrocene compounds with similar chemical and physical characteristics, yet covering a broad range of electrochemical potentials should circumvent this problem. To increase the ferrocene/ferrocenium potential, electron-withdrawing substituents are required. Mono-, 1,1’-diesters and a single 1,1’,3-triester of ferrocene are known [45–53]. Elegant routes to 1,1’,3-tris(methoxycarbonyl)ferrocene and 1,1’,3,3’-tetrakis(methoxycarbonyl)ferrocene were developed only very recently [54], complementing the series of methyl esters of ferrocene carboxylic acids **1**–**4** (Scheme 2) [45–52].

The extremely bulky and electron-poor pentakis(methoxycarbonyl)cyclopentadienyl ligand gives a pseudo octahedral high-spin iron(II) complex **5**, instead of forming a stable classical low-spin sandwich complex, precluding its application as redox mediator (Scheme 2) [55–56].

Ferrocenyl esters **1**–**4** are synthetically accessible via the acids of **1** [45–46], **2** [57], **3** and **4** [54] in a direct selective metalation of ferrocene [54,57–60], quenching with carbon dioxide, followed by esterification [45–48,54]. The 1,1’-disubstituted ferrocene **2** can also be obtained by direct coordination of the respective substituted cyclopentadienyl ligand (CpR) to iron(II) [49]. An alternative route to the mono-, 1,1’-di- and 1,1’,3-tricarboxylic acids of ferrocene is the oxidation of the respective acetylferrocenes [47–48,53]. Ferrocene carboxylic acid is also available via basic hydrolysis of ferrocenyl aryl ketones [61]. Together with the redox potentials of ferrocene, **1** and **2**, the hitherto unknown electrochemical potentials of **3** and **4** should cover a wide potential range. This will meet the requirements of different substrates for the potential application of **1**–**4** and their ferrocenium ions as selective redox mediators or SET reagents. Apart from the redox potentials of the redox mediators FcH and **1**–**4**, the stability of the 18 and 17 valence electron species as well as their solubility and the availability of spectroscopic probes to monitor reaction progress and stability are important issues. These fundamental aspects will be addressed in this study.

Herein, a detailed study of the properties of **1**–**4** and their ferrocenium ions **1^+^**–**4^+^** in solution is reported including electrochemical methods (cyclic voltammetry and square wave voltammetry; CV, SWV) and covering investigations regarding the stability of **1**–**4**/**1^+^**–**4^+^** by spectroelectrochemical methods (UV–vis, IR) [62–68]. In addition, the mediators **1**/**1^+^**–**4**/**4^+^** are probed by paramagnetic NMR spectroscopic methods [69–73]. The results are supported with (time-dependent) density functional theoretical (TD)-DFT methods.

## Results and Discussion

### Electrochemistry of esters **1**–**4**

**Figure 1:**
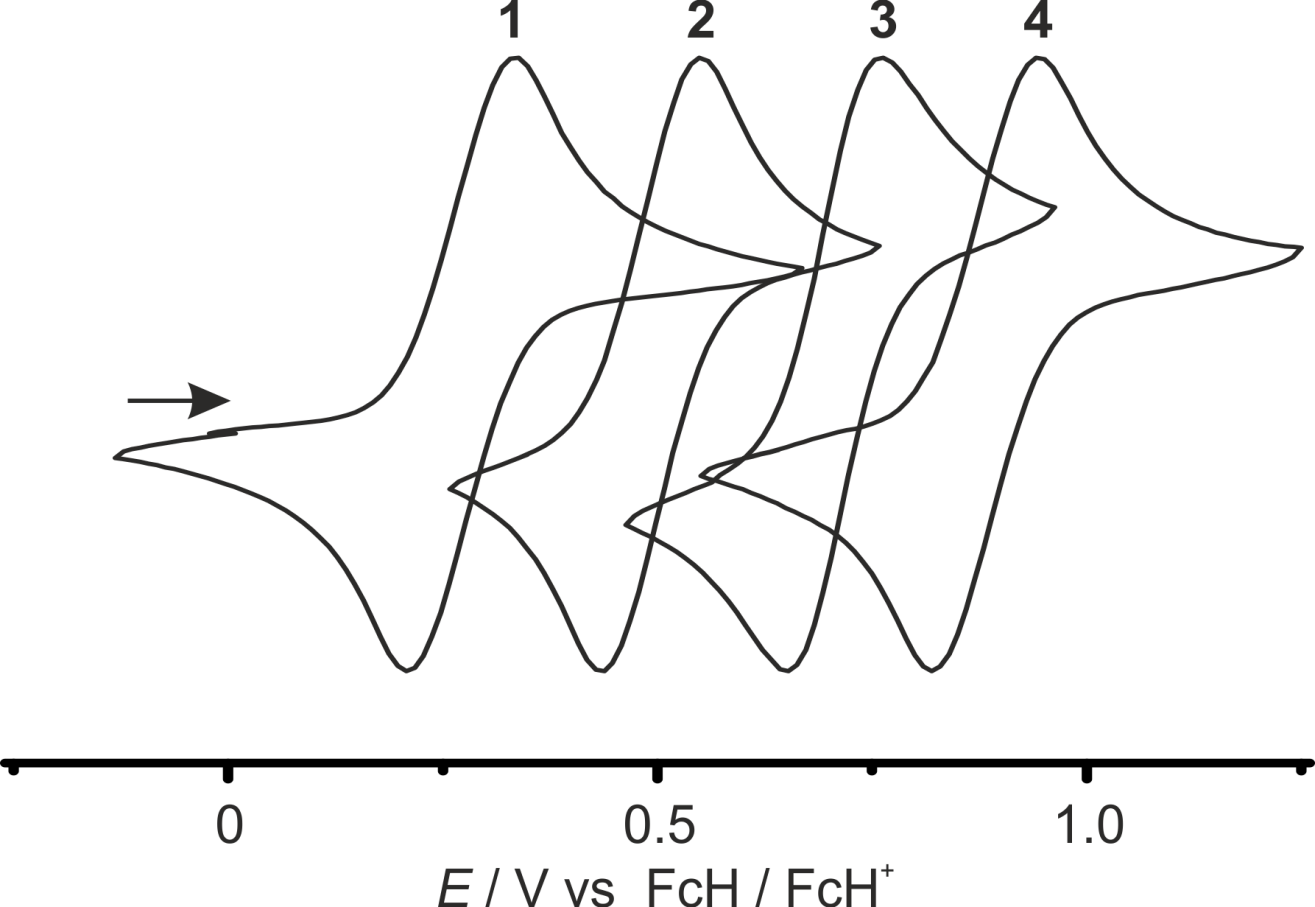
Normalized cyclic voltammograms for anodic sweeps of **1**–**4** in CH_2_Cl_2_/[*n-*Bu_4_N][B(C_6_F_5_)_4_] (scan rate 100 mV s^−1^).

The esters **1**–**4** were studied by cyclic and square wave voltammetry in 0.1 M CH_2_Cl_2_ solutions of [*n-*Bu_4_N][B(C_6_F_5_)_4_], using platinum working and counter electrodes. All esters **1**–**4** show an essentially reversible behaviour for the ferrocene/ferrocenium oxidation process (Figure 1, Figure S1, Supporting Information File 1). The electrochemical potentials cover a wide range, *E*_1/2_ = 260–900 mV vs FcH/FcH^+^ (Figure 1, Table 1).

**Table 1:** Electrochemical data of esters **1**–**4** and sum of Hammett substituent constants σ_p_^a^ and σ_m_^a^.

	*E*_1/2_ [mV]^b^	∑σ_p/m_	∑σ_p_

**1**	260	σ_p_ = 0.45	0.45
**2**	495	2 σ_p_ = 0.90	0.90
**3**	700	2 σ_p_ + σ_m_ = 1.27	1.35
**4**	900	2 σ_p_ + 2 σ_m_ = 1.64	1.80

^a^σ_p_ = 0.45, σ_m_ = 0.37 for COOMe substituent [74]. ^b^vs FcH/FcH^+^.

The oxidation potential of the tetraester **4** is very high with *E*_1/2_ = 900 mV. To the best of our knowledge, higher oxidation potentials (vs FcH/FcH^+^) have been observed only for 1,1’,2,2’,4,4’-hexakis(pentafluorophenyl)ferrocene (940 mV in CH_2_Cl_2_) [40], 1,1’,2,2’,3,3’-hexakis(pentafluorophenyl)ferrocene (951 mV in CH_2_Cl_2_) [40], decachloroferrocene (*E*_p_ = 1246 mV in MeCN) [37], 1,1’,2-tri(formyl)ferrocene (910 mV in CH_2_Cl_2_ at −40 °C) [38] and 1,1’,2,2’-tetra(formyl)ferrocene (1145 mV in CH_2_Cl_2_ at −40 °C) [38]. The latter three are only irreversibly oxidized at room temperature precluding any application as mediators. The data are in full accordance with the increasing electron-withdrawing character of the cyclopentadienyl ligands from **1** to **4**. The position of the ester groups has a slight influence on the electrochemical potential. 1- or 1’-substitution with a methoxycarbonyl group raises the potential by ca. 250 mV (FcH → **1**, **1** → **2**), while substitution in 3- and 3’-position has only a smaller impact with an increase of the potential by ca. 200 mV (**2** → **3**, **3** → **4**). According to Lever et al. [39], the calculated electrochemical parameters *E*_L_(L) for 1-(methoxycarbonyl)cyclopentadienyl and 1,3-bis(methoxycarbonyl)cyclopentadienyl ligands amount to *E*_L_(L_1_) = 250 mV and *E*_L_(L_2_) = 450 mV vs FcH/FcH^+^, respectively. Indeed, the electrochemical potential *E*_1/2_ = 700 mV of **3** perfectly corresponds to the sum *E*_L_(L_1_) + *E*_L_(L_2_) = 700 mV. Consequently, the ligand contributions to the electrochemical potential of substituted cyclopentadienyl complexes are essentially additive for **1**–**4**.

This characteristic relationship is supported by correlating the electrochemical data with the Hammett substituent constants [37,39,74–75]. Typically, the *E*_1/2_ data of substituted ferrocenes correlate linearly with the sum ∑σ_p_ of the Hammett values σ_p_ of *para*-substituents [37,39,74].

**Figure 2:**
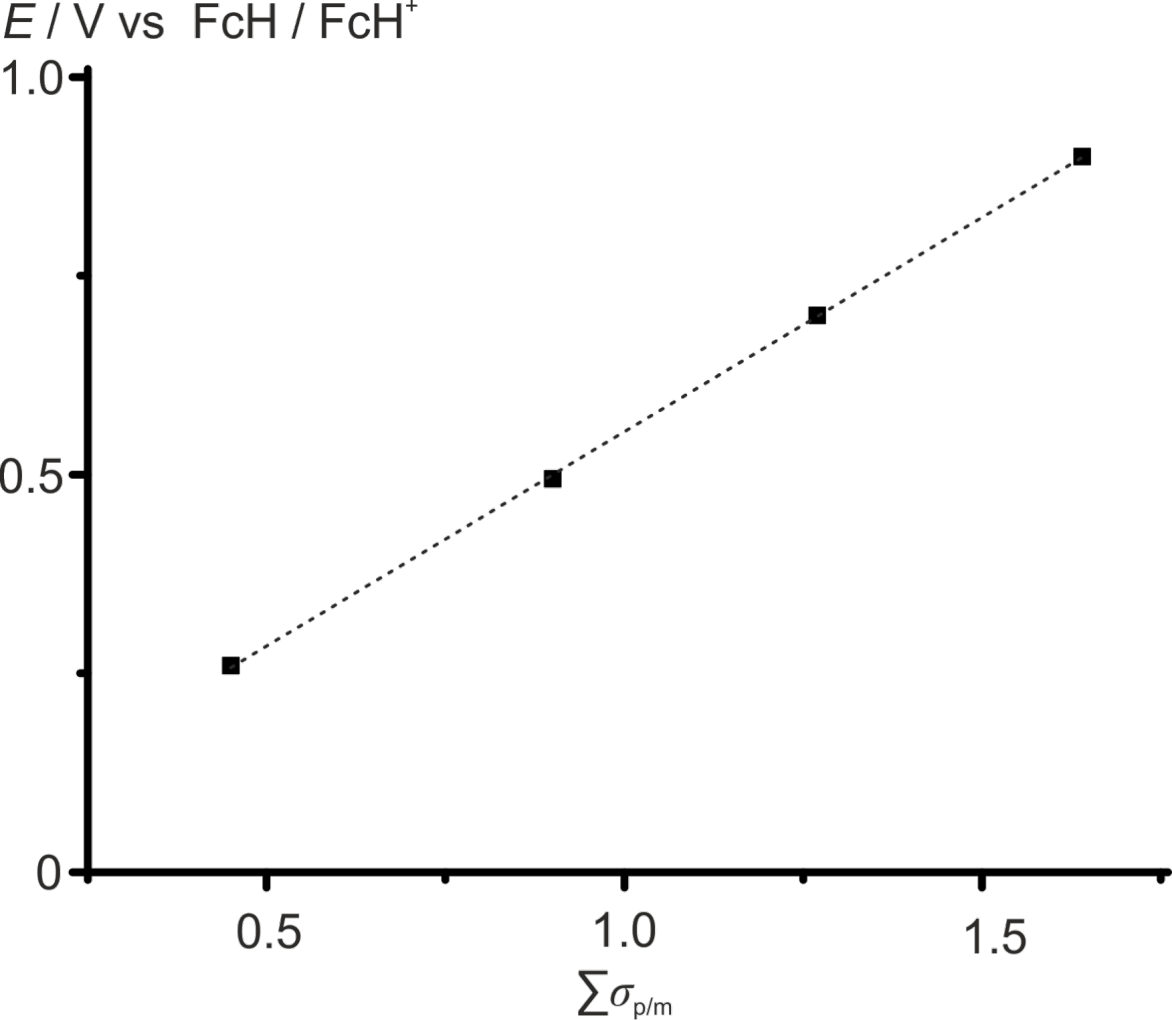
Electrochemical potentials *E*_1/2_ (vs FcH/FcH^+^) of esters **1**–**4** versus sum of Hammett values ∑σ_p/m_ with linear regression (*E*_1/2_ = 0.539 V ∙ ∑σ_p/m_ + 0.015 V, *R*^2^ = 0.9999).

For esters **1**–**4**, the electrochemical potentials *E*_1/2_ (vs FcH/FcH^+^) versus sum of Hammett values ∑σ_p_ did not give a satisfactory linear relation. Within this approach, the relative positions of ester groups and hence their different electronic influence to the electrochemical potential is not considered. The influence of a methoxycarbonyl substituent in 1- or 1’-position is indeed best described with σ_p_ = 0.45 [75]. On the other hand, substituents in the 3- or 3’-position require using σ_m_ = 0.37 [75] for *meta*-substituents, to give an excellent linear correlation of *E*_1/2_ with ∑σ_p/m_ (Figure 2, Table 1).

The generalizable use of σ_p_ and especially σ_m_ to include the effect on the relative positions of substituents for *E*_1/2_ of polysubstituted ferrocenes has to be further validated with other series of polysubstituted ferrocenes.

### IR spectroelectrochemistry of esters **1**–**4**

**Figure 3:**
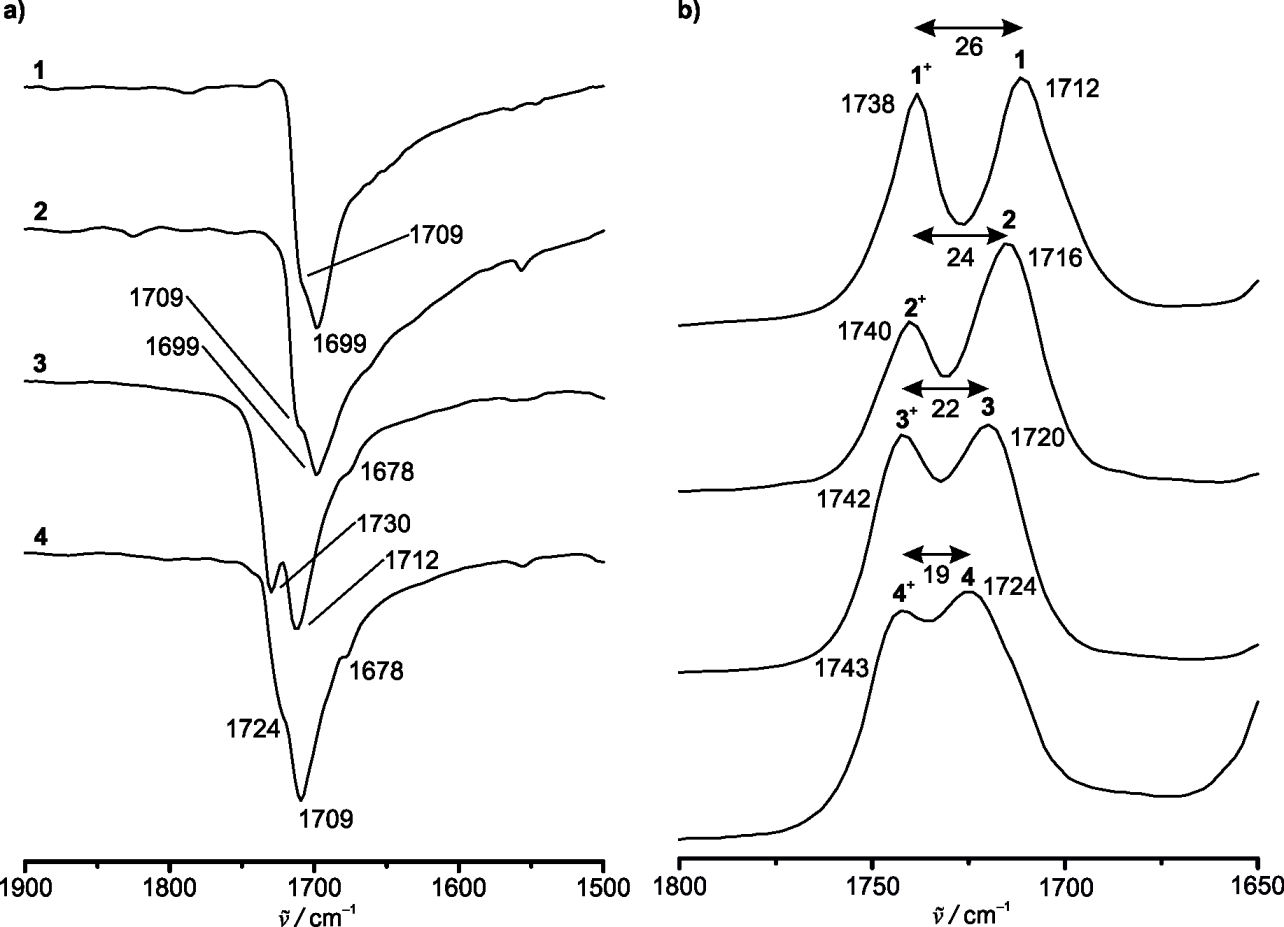
a) Partial ATR IR spectra (transmission normalized, C=O stretching vibration region) of solids **1**–**4**. b) Partial spectroelectrochemical IR spectra (absorption normalized, C=O stretching vibration region) of mixtures of the **1**/**1^+^**–**4**/**4^+^** redox couples in dichloromethane/[*n-*Bu_4_N][B(C_6_F_5_)_4_].

In the attenuated total reflection (ATR) IR spectra of solid samples of esters **1**–**4**, several overlapping bands for the C=O stretching vibrations of the ester substituents are observed between 1678 and 1730 cm^−1^ (Figure 3a, Figures S2–S6, Table S1, Supporting Information File 1). DFT calculations (B3LYP, def2-TZVP, RIJCOSX, ZORA, CPCM (CH_2_Cl_2_)) on di-, tri- and tetraesters **2**–**4** suggest an intramolecular coupling of the C=O vibrations of the ester moieties substantiating the number of observed bands (Table S1, Supporting Information File 1). Furthermore, crystal packing effects with intermolecular C=O∙∙∙H–C interactions, differing in strengths, can be responsible for the occurrence of distinguishable C=O bands [54,76–77]. For example, two different molecules of monoester **1** are present in the asymmetric unit of the solid-state structure [76], leading to different C=O stretching vibration bands (Figure 3a).

In contrast to the solid-state IR spectra, only a single broad C=O band is observed for **1**–**4** in solution (Figure 3, Figure 4, Figures S7–S14, Supporting Information File 1). In the series **1**–**4**, the C=O bands shift to higher wavenumbers in solution 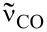 = 1712–1724 cm^−1^ with increasing number of electron-withdrawing COOMe groups (Figure 3b). The DFT calculated IR spectra with unscaled energies of the C=O vibrations 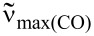 = 1710–1724 cm^−1^ fully support these findings (Table S1, Figures S15–S22, Supporting Information File 1).

**Figure 4:**
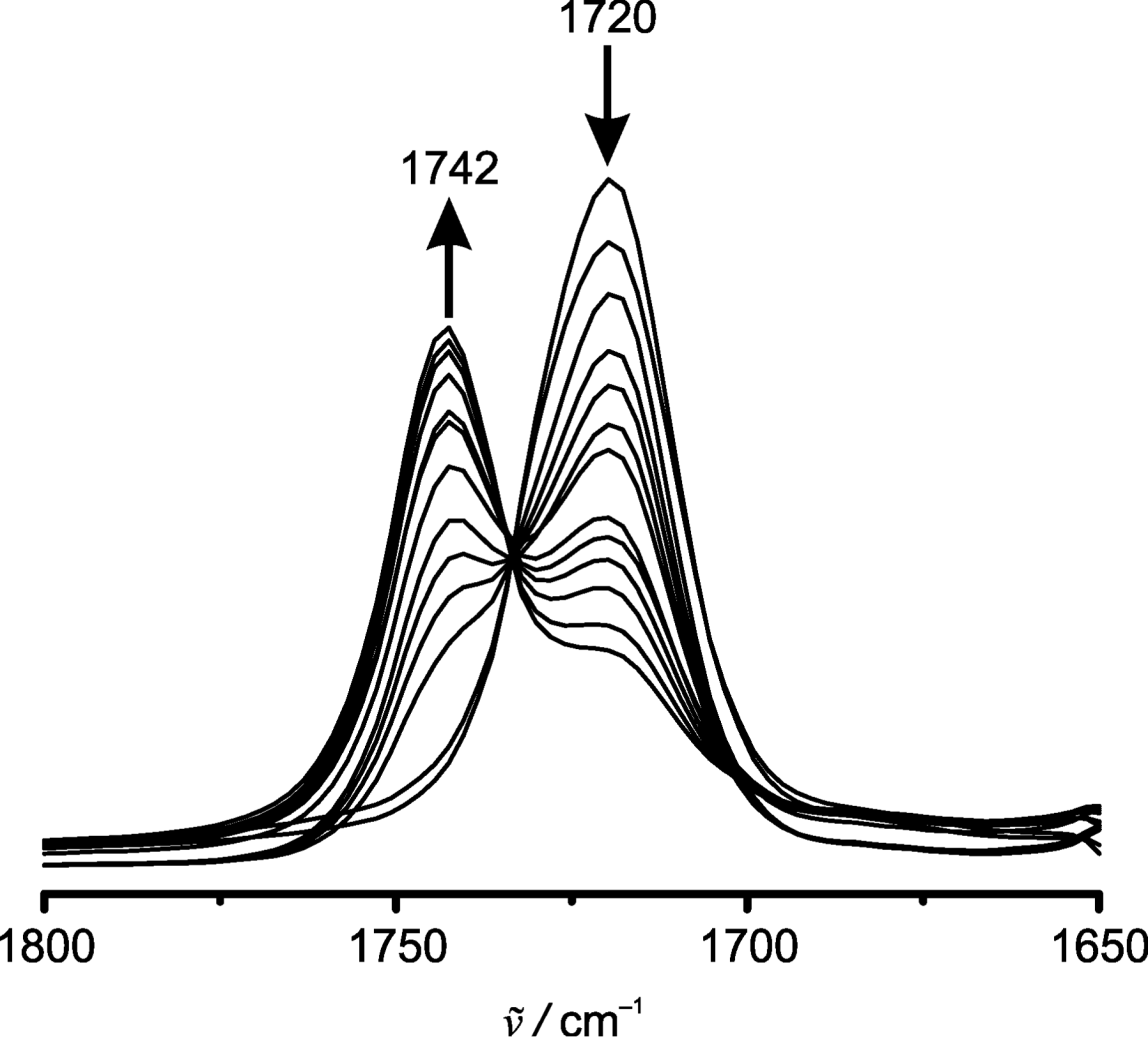
IR spectroelectrochemical oxidation of **3** to **3^+^** in CH_2_Cl_2_/[*n-*Bu_4_N][B(C_6_F_5_)_4_] (C=O stretching vibration region, 0.4–1.1 V vs Ag pseudo reference electrode).

Compounds **1**–**4** can be reversibly oxidized to **1^+^**–**4^+^** in dichloromethane and [*n-*Bu_4_N][B(C_6_F_5_)_4_] as supporting electrolyte in an IR spectroelectrochemical (SEC) cell, confirming the chemical stability of the ferrocenyl esters under the conditions of electrolysis (Figure 3b, Figure 4, Figures S7–S14, Table S1, Supporting Information File 1).

Triester **3** and tetraester **4** cannot be quantitatively oxidized to **3^+^** and **4^+^** in the SEC cell up to a potential of 1.1 V and 1.4 V, respectively, probably due to a fast diffusion of **3** and **4** to the anode in the beam path (Figure 4, Figures S11–S14, Supporting Information File 1). In addition, precipitation of some poorly soluble [**4**][**X**] also occurs. During oxidation to the respective ferrocenium cations, the C=O stretching vibration bands of **1**–**4** decrease in intensity, while the C=O bands of **1^+^**–**4^+^** appear, crossing in clean isosbestic points. Expectedly, the C=O stretching vibrations of **1^+^**–**4^+^** are shifted to higher wavenumbers by 26–19 cm^−1^ (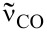 = 1738–1743 cm^–1^) with an increasing electron-withdrawing character of the Cp ligands. The substituent effect is attenuated by the positive charge at the iron atom in **1^+^**–**4^+^** (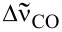 = 5 cm^−1^), compared to **1**–**4** (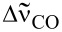 = 12 cm^−1^), respectively (Figure 3b, Table S1, Figures S7–S14, Supporting Information File 1) [78]. The unscaled energies of the DFT calculated C=O bands of **1^+^**–**3^+^** fit very well to the experimental observations of **1^+^**–**3^+^** (Figures S7–S12, S16, S18, S20, Table S1, Supporting Information File 1). Unexpectedly, the calculated 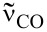 data of **4^+^** are significantly lower than the experimental ones, which remain unexplained at the moment.

For all redox couples of the ferrocenyl esters, the C=O stretching vibration delivers a useful in operando probe substantiating the stability of the redox mediator and enabling quantification of both redox partners and hence estimation of the actual concentration-dependent redox potential in solution.

### UV–vis spectroelectrochemistry of esters **1**–**4**

**Figure 5:**
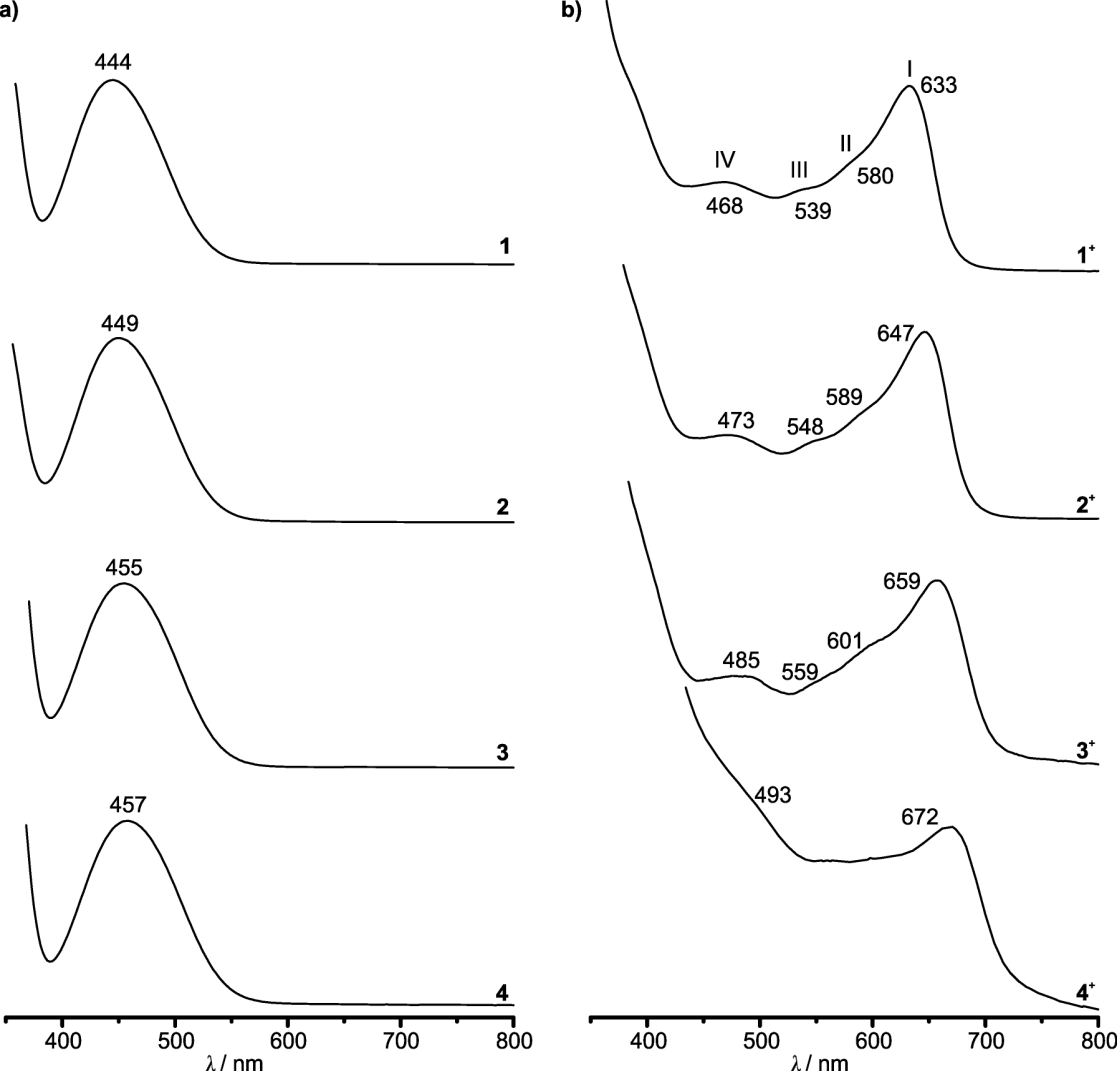
a) Normalized UV–vis spectra of **1**–**4** in CH_2_Cl_2_. b) Normalized UV–vis absorptions of **1^+^**–**4^+^** in CH_2_Cl_2_/[*n-*Bu_4_N][B(C_6_F_5_)_4_] after spectroelectrochemical oxidation of **1**–**4**.

Analogous to the IR-SEC experiments, the esters **1**–**4** were also probed by UV–vis-SEC investigations. The UV–vis spectra of **1**–**4** recorded in dichloromethane show the ferrocene ligand field absorption band at λ_max_ = 444, 449, 455 and 457 nm, which is typically around λ_max_ ≈ 440–490 nm [66,70,79–80] (Figure 5a).

**Figure 6:**
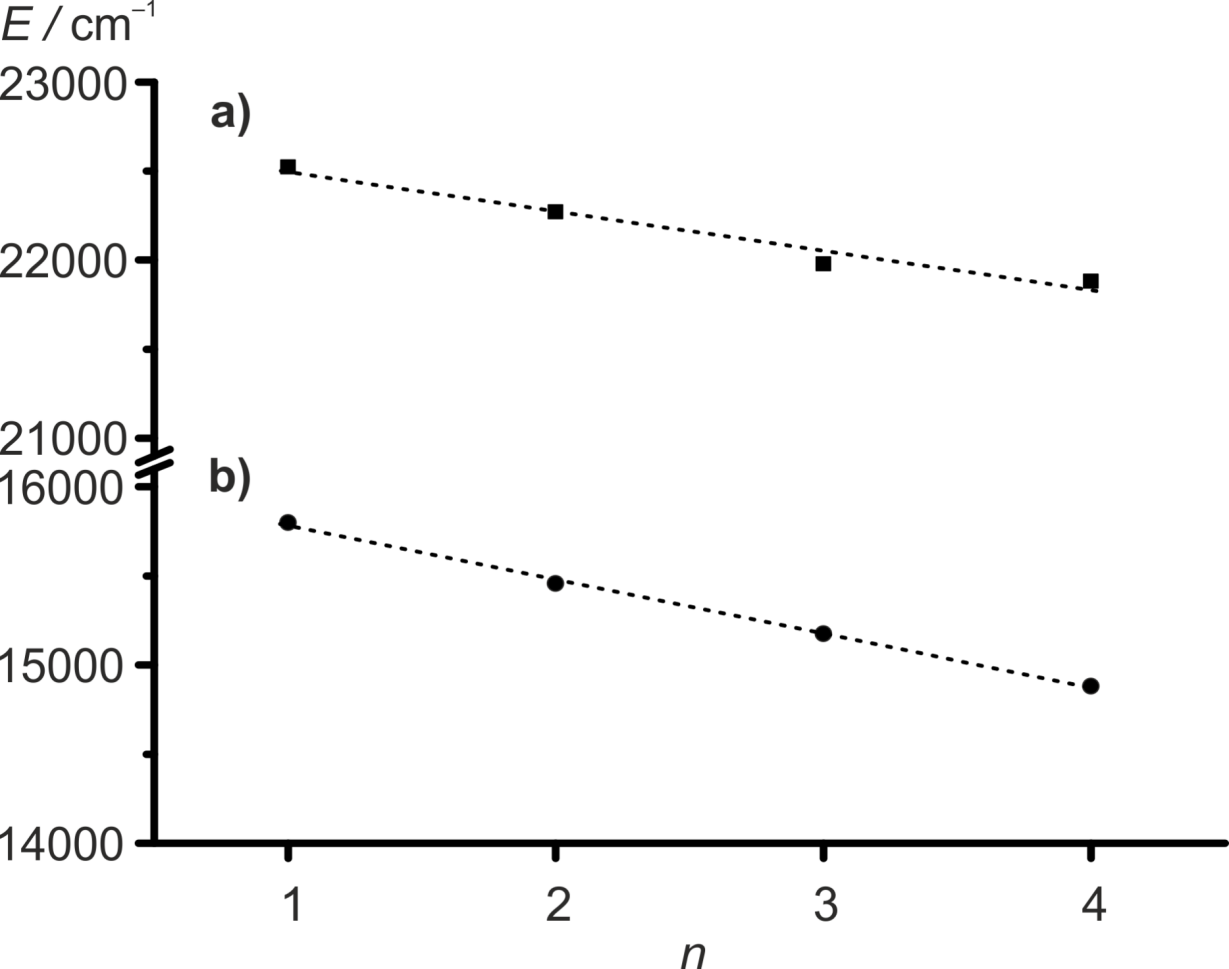
Absorption energy *E* of ferrocene bands of **1**–**4** (a) and energies of the low energy absorption maxima at around λ_max_ ≈ 630–670 nm of **1^+^**–**4^+^** (b) versus number of methoxycarbonyl substituents *n* with regression lines a) *E* = −222 cm^−1^ ∙ *n* + 22700 cm^−1^, *R*^2^ = 0.9824; b) *E* = −303 cm^−1^ ∙ *n* + 16100 cm^−1^, *R*^2^ = 0.9991.

The energy of the absorption bands decreases almost linearly with the number *n* of the electron-withdrawing COOMe substituents for **1**–**4** (Figure 6a).

The reversible oxidation of **1**–**4** in UV–vis-SEC experiments in CH_2_Cl_2_/[*n-*Bu_4_N][B(C_6_F_5_)_4_] is monitored by the decreasing band intensity of the ferrocene absorption and the appearance of a set of four partially resolved characteristic ferrocenium absorptions (bands I–IV) responsible for the blue color (Figure 5b and Figure 7, Figures S23–S31, Supporting Information File 1).

**Figure 7:**
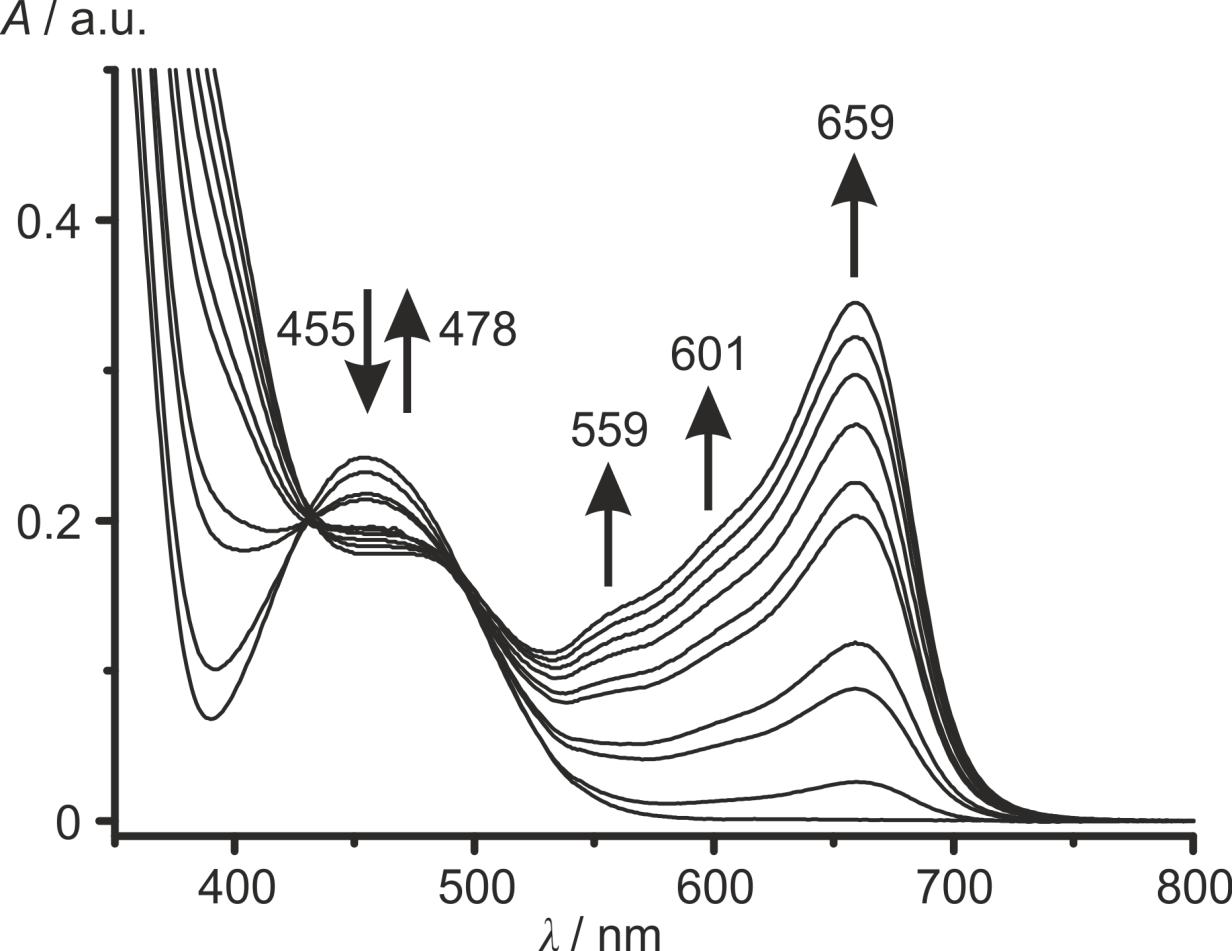
UV–vis spectroelectrochemical oxidation of **3** in CH_2_Cl_2_/[*n*-Bu_4_N][B(C_6_F_5_)_4_] (0–1.1 V vs Ag pseudo reference electrode).

Isosbestic points indicate clean conversions of **1** → **1^+^**, **2** → **2^+^** and **3** → **3^+^**, respectively. For example, this set of bands and shoulders (sh) IV–I is observed at λ_max_ = 485 nm (IV), λ_sh_ = 559 nm (III), 601 nm (II) and λ_max_ = 659 nm (I) for **3^+^**. During oxidation of **4** to **4^+^**, isosbestic points between the absorption bands of **4** and **4^+^** cannot be observed (Figures S29 and S30, Supporting Information File 1). Probably, precipitation of the poorly soluble tetraester **4^+^** could be responsible for this effect, as already suggested for the IR-SEC experiments of **4**/**4^+^**. On the other hand, isosbestic points are observed in the UV–vis spectra upon re-reduction of **4^+^** to **4** (Figure S31, Supporting Information File 1). The energy of the absorptions of the ferrocenium cations **1^+^**–**4^+^** decreases with the electron-withdrawing nature of the Cp ligands in the series **1^+^**–**4^+^**, similar to the vis absorption maxima of the neutral ferrocenes **1**–**4**. For the prominent band I of the cations **1^+^**–**4^+^**, a linear and stronger dependency of the energy on the number *n* of methoxycarbonyl substituents can be found than for the ligand field band of the ferrocenes **1**–**4** (Figure 6b). The lowest energy band (band I) in the UV–vis spectra of **1^+^**–**4^+^** is assigned to ligand-to-metal charge transfer (LMCT) transitions [79,81–83]. The bands II–IV are assigned to mainly d–d transitions [79]. TD-DFT calculations on the B3LYP, def2-TZVP, RIJCOSX, ZORA, CPCM (CH_2_Cl_2_) level do not give satisfactory results concerning energy, number of bands and oscillator strength of electronic transitions (Figures S32–S35, Supporting Information File 1). The poor agreement of TD-DFT calculated electronic spectra of metallocenes and derivatives with experimental data has been noted before. Improvements have been achieved by testing different functionals [84–85] and by including vibrational distortions of the ferrocene geometry into the calculations [86]. Nevertheless, the LMCT character of the prominent band I is confirmed by the calculations. The intensity of band I scales with the amount of the corresponding ferrocenium ion present and consequently the actual potential in solution can be estimated by UV–vis spectroscopy.

### NMR spectroscopy of esters **1**–**4** and **1^+^**–**4^+^**

In contrast to typical organic paramagnetic redox mediators, the relaxation properties of proton nuclei of paramagnetic ferrocenium derivatives allow the observation of reasonable sharp resonances [87]. The fast electron self-exchange of the ferrocene/ferrocenium redox couple and derivatives on the NMR timescale leads to the observation of resonances with averaged chemical shifts δ in the ^1^H NMR spectra of ferrocene/ferrocenium mixtures [5–6,70–72]. The molar fraction of FcH/FcH^+^ can be calculated from the averaged ^1^H NMR resonance frequencies of a mixture and the known resonance frequencies of FcH and FcH^+^, respectively [6]. This relation gives χ_P_ = (δ − δ_D_)/( δ_P_ − δ_D_) for the molar fraction of the paramagnetic species, expressed in the chemical shift scale with δ_D_ being the chemical shift of the diamagnetic species, δ_P_ being the resonance of the paramagnetic species and δ being the averaged chemical shift of the mixture.

**Figure 8:**
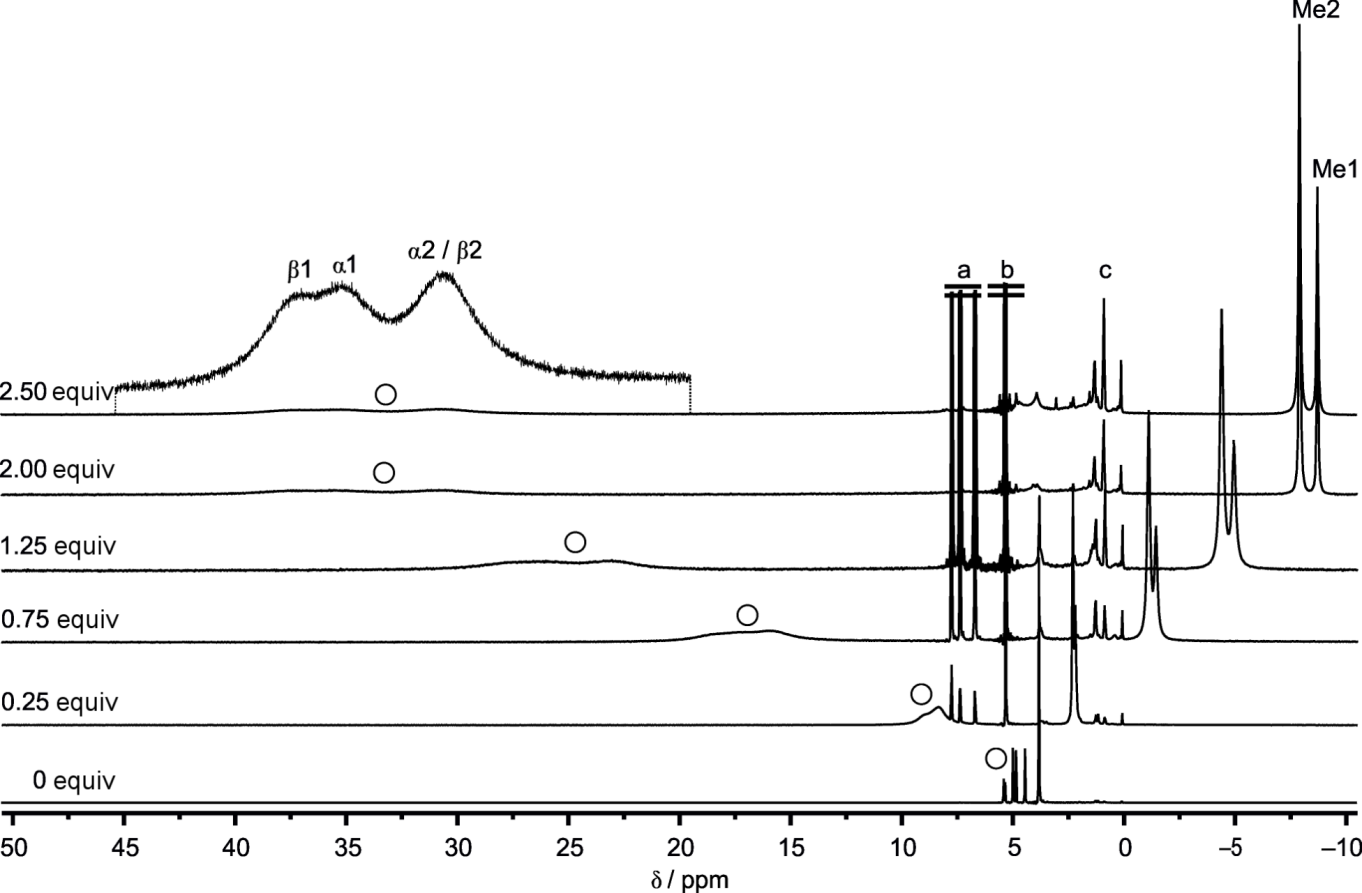
^1^H NMR oxidation titration of **3** in CD_2_Cl_2_ with [N(2,4-C_6_H_3_Br_2_)_3_]^+^ as oxidant. ^a^[N(2,4-C_6_H_3_Br_2_)_3_]. ^b^CDHCl_2_. ^c^Residual solvents and grease.

The detection of the resonances of **1**/**1^+^**–**4**/**4^+^** should allow for determining the ratio of **1**:**1^+^**–**4**:**4^+^** by in situ NMR experiments. Thus, titration of **1**–**4** with Magic Green, tris(2,4-dibromophenyl)ammoniumyl hexachloroantimonate [10], as a strong oxidant (*E*_1/2_ = 1140 mV in MeCN vs FcH/FcH^+^) in CD_2_Cl_2_ under NMR monitoring shows that the Cp proton resonances broaden upon oxidation and shift to lower field, while the methyl proton resonances of the ester substituents shift to higher field and remain much sharper (Figure 8, Table 2, Figures S36–S38, Supporting Information File 1).

**Table 2:** ^1^H NMR data (δ [ppm])^a^ of **1**–**4** and **1^+^**–**4^+^** in CD_2_Cl_2_.

	H^Cp^	H^α1^/H^β1^	H^α2^/H^β2^	H^Me1^	H^Me2^

**1**	4.20	4.40/4.77		3.76	
**1^+^**	37.0	30.1/32.3		−8.34	
**2**		4.42/4.79		3.78	
**2^+^**		34.0^b^		−8.51	
**3**		4.43/4.84	4.97/5.39	3.79	3.80
**3^+^**		35.3/37.5	30.7^b^	−8.75	−7.94
**4**			4.98/5.42		3.87
**4^+^**			33.6^b^		−8.26

^a^Numbering scheme: 
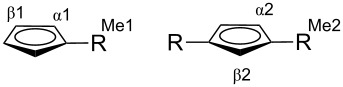
. ^b^Only a single broad resonance.

In some cases, e.g., **3^+^**, the different Cp protons can still be distinguished in spite of the broadened resonances (Figure 8). The broadening is much more severe for the Cp proton resonances, while the methyl proton resonances are still rather sharp allowing the discrimination and assignment of the different methyl protons of **3^+^** (Figure 8).

With an increasing number of ester groups, the proton resonances of the mono- and disubstituted Cp ligands and of the methyl groups shift to lower field for **1**–**4** (CpR: **1** → **2** → **3**, CpR_2_: **3** → **4**), while for **1^+^**–**4^+^**, the Cp ligand proton resonances shift to lower field and the methyl proton resonances shift to higher field (CpR: **1^+^** → **2^+^** → **3^+^**, CpR_2_: **3^+^** → **4^+^**). This substituent effect is larger for the paramagnetically shifted resonances of **1^+^**–**4^+^** than for the diamagnetic complexes **1**–**4**.

In CD_3_CN, the treatment of **3** with Magic Green led to the disappearance of the resonances of **3**. However, paramagnetically shifted resonances of **3^+^** are absent suggesting that the initially formed **3^+^** undergoes further reactions with the coordinating solvent CD_3_CN (Figure S39, Supporting Information File 1). This finding underscores that the solvent has to be carefully chosen with respect to the mediated reaction and stability of the mediator.

From the observed ^1^H NMR chemical shifts – either of the cyclopentadienyl or methyl resonances – the relative concentrations of the ferrocene and ferrocenium ion can be extracted, again allowing the estimation of the actual potential in solution by spectroscopic techniques.

## Conclusion

Ferrocenyl esters **1**–**4** with one to four ester substituents are reversibly oxidized to the respective ferrocenium cations **1^+^**–**4^+^**, spanning a broad electrochemical potential range from 260 mV for **1** to 900 mV for **4** vs the ferrocene/ferrocenium redox couple. The electrochemical potentials *E*_1/2_ of **1**–**4** correlate linearly with the sum of Hammett substituent parameters ∑σ_p/m_. However, the position of ester substituents has to be taken into account by employing σ_p_ for 1- and 1’-substituents and σ_m_ for 3- and 3’-substituents, respectively. Complexes **1**–**4** and **1^+^**–**4^+^** are stable under conditions of electrolysis (CH_2_Cl_2_, [*n-*Bu_4_N][B(C_6_F_5_)_4_]) as demonstrated by IR and UV–vis spectroelectrochemical experiments and ^1^H NMR spectroscopy. The C=O stretching vibrations of the ester substituents as characteristic probes in the IR spectra are consistently shifted to higher energies from **1** to **4** and from **1^+^** to **4^+^**. Upon oxidation of **1**–**4** to **1^+^**–**4^+^** in solution, the ferrocene bands in the UV–vis spectra of **1**–**4** at λ_max_ = 444–457 nm and the LMCT bands of **1^+^**–**4^+^** at λ_max_ = 633–672 nm bathochromically shift linearly with increasing number of ester groups. The ^1^H NMR paramagnetic chemical shifts of **1^+^**–**4^+^** have been determined by redox titration experiments.

With all the data in hand, the molar fraction of the ester-substituted redox couples **1**/**1^+^**–**4**/**4^+^** can be accessed a) from the C=O stretching vibrations of the ester groups, b) the ferrocenium CT bands or c) from the averaged ^1^H NMR chemical shifts of the Cp or ester methyl protons. Ongoing investigations focus on the spectroscopic monitoring of **1–4** as redox mediators in selected electrosynthetic transformations.

## Experimental

Dichloromethane, CD_2_Cl_2_ and CD_3_CN were distilled from calcium hydride. Electrochemical experiments were carried out on a BioLogic SP-50 voltammetric analyzer using a platinum working electrode, a platinum wire as counter electrode, and a 0.01 M Ag/AgNO_3_ CH_3_CN electrode as reference electrode. The measurements were carried out at a scan rate of 100 mV s^−1^ for cyclic voltammetry experiments and 100 mV s^−1^ for square wave voltammetry experiments using 0.1 M [*n-*Bu_4_N][B(C_6_F_5_)_4_] as supporting electrolyte and 0.001 M of the sample in dichloromethane. Potentials are given relative to the ferrocene/ferrocenium couple.

Spectroelectrochemical experiments were performed using a Specac omni-cell liquid transmission cell with CaF_2_ windows equipped with a Pt-gauze working electrode, a Pt-gauze counter electrode and an Ag wire as pseudo-reference electrode, melt-sealed in a polyethylene spacer (approximate path length 0.5 mm) in dichloromethane (68, 35, 13, 2 mM solutions of **1**–**4** in CH_2_Cl_2_, containing 0.1 M [*n-*Bu_4_N][B(C_6_F_5_)_4_]) [88]. UV–vis/near-IR spectra were recorded on a Varian Cary 5000 spectrometer using 1.0 cm cells (Hellma, Suprasil). IR spectra were recorded on a Bruker Alpha FTIR spectrometer with ATR unit, containing a diamond crystal.

NMR spectra were recorded on a Bruker Avance DRX 400 spectrometer at 400.31 MHz (^1^H) at 25 °C. All resonances are reported in ppm versus the solvent signal as internal standard: CD_2_Cl_2_ (^1^H, δ = 5.32 ppm), CD_3_CN (^1^H, δ = 1.94 ppm) [89].

DFT calculations were carried out using the ORCA program package (version 4.0.1) [90]. All calculations were performed using the B3LYP functional [91–93] and employ the RIJCOSX approximation [94–95]. Relativistic effects were calculated at the zeroth order regular approximation (ZORA) level [96]. The ZORA keyword automatically invokes relativistically adjusted basis sets. To account for solvent effects, a conductor-like screening model (CPCM) modeling dichloromethane was used in all calculations [97]. Geometry optimizations and TD-DFT calculations (50 vertical transitions) were performed using Ahlrichs’ split-valence triple-ξ basis set def2-TZVP which comprises polarization functions for all non-hydrogen atoms [98–99]. The presence of energy minima was checked by numerical frequency calculations. Explicit counterions and/or solvent molecules were not taken into account.

## Supporting Information

The Supporting Information file contains square wave voltammograms, IR and UV–vis spectra of the spectroelectrochemical experiments, (TD)-DFT calculated IR and UV–vis spectra, a table with IR data, ^1^H NMR spectra of the oxidation titration experiments and Cartesian coordinates of DFT calculated structures of **1**–**4**.

File 1 Mediators measured and calculated spectra, IR data and Cartesian coordinates.

## References

[1] Togni A, Hayashi T (1994). Ferrocenes.

[2] Štěpnička P (2008). Ferrocenes. Ligands, materials and biomolecules.

[3] Astruc D (2017). Eur J Inorg Chem.

[4] Pavlishchuk V V, Addison A W (2000). Inorg Chim Acta.

[5] Yang E S, Chan M-S, Wahl A C (1980). J Phys Chem.

[6] Nielson R M, McManis G E, Safford L K, Weaver M J (1989). J Phys Chem.

[7] Gagne R R, Koval C A, Lisensky G C (1980). Inorg Chem.

[8] Gritzner G, Kuta J (1984). Pure Appl Chem.

[9] Noviandri I, Brown K N, Fleming D S, Gulyas P T, Lay P A, Masters A F, Phillips L (1999). J Phys Chem B.

[10] Connelly N G, Geiger W E (1996). Chem Rev.

[11] Steckhan E (1986). Angew Chem, Int Ed Engl.

[12] Francke R, Little R D (2014). Chem Soc Rev.

[13] Wiebe A, Gieshoff T, Möhle S, Rodrigo E, Zirbes M, Waldvogel S R (2018). Angew Chem, Int Ed.

[14] Jiang Y, Xu K, Zeng C (2018). Chem Rev.

[15] Ding Y, Yu G (2017). Angew Chem, Int Ed.

[16] Raoof J B, Ojani R, Karimi-Maleh H, Hajmohamadi M R, Biparva P (2011). Anal Methods.

[17] Jahn U (1998). J Org Chem.

[18] Langer T, Illich M, Helmchen G (1996). Synlett.

[19] Jahn U, Müller M, Aussieker S (2000). J Am Chem Soc.

[20] Jahn U, Hartmann P, Dix I, Jones P G (2001). Eur J Org Chem.

[21] Pigge F C, Coniglio J J, Rath N P (2004). J Org Chem.

[22] Sibi M P, Hasegawa M (2007). J Am Chem Soc.

[23] Goddard J-P, Gomez C, Brebion F, Beauvière S, Fensterbank L, Malacria M (2007). Chem Commun.

[24] Richter J M, Whitefield B W, Maimone T J, Lin D W, Castroviejo M P, Baran P S (2007). J Am Chem Soc.

[25] Krygowski E S, Murphy-Benenato K, Shair M D (2008). Angew Chem, Int Ed.

[26] Jahn U, Dinca E (2009). Chem – Eur J.

[27] Khobragade D A, Mahamulkar S G, Pospíšil L, Císařová I, Rulíšek L, Jahn U (2012). Chem – Eur J.

[28] Holan M, Pohl R, Císařová I, Klepetářová B, Jones P G, Jahn U (2015). Chem – Eur J.

[29] Chernyak N, Buchwald S L (2012). J Am Chem Soc.

[30] Marciasini L D, Richy N, Vaultier M, Pucheault M (2013). Adv Synth Catal.

[31] Foo K, Sella E, Thomé I, Eastgate M D, Baran P S (2014). J Am Chem Soc.

[32] Hernández-Muñoz L S, Fragoso-Soriano R J, Vázquez-López C, Klimova E, Ortiz-Frade L A, Astudillo P D, González F J (2010). J Electroanal Chem.

[33] Hernández-Muñoz L S, Galano A, Astudillo-Sánchez P D, Abu-Omar M M, González F J (2014). Electrochim Acta.

[34] Hou Z-W, Mao Z-Y, Zhao H-B, Melcamu Y Y, Lu X, Song J, Xu H-C (2016). Angew Chem, Int Ed.

[35] Wu Z-J, Xu H-C (2017). Angew Chem, Int Ed.

[36] Zhu L, Xiong P, Mao Z-Y, Wang Y-H, Yan X, Lu X, Xu H-C (2016). Angew Chem, Int Ed.

[37] Brown K N, Gulyas P T, Lay P A, McAlpine N S, Masters A F, Phillips L (1993). J Chem Soc, Dalton Trans.

[38] Hildebrandt A, Khalyfeh K A, Schaarschmidt D, Korb M (2016). J Organomet Chem.

[39] Lu S, Strelets V V, Ryan M F, Pietro W J, Lever A B P (1996). Inorg Chem.

[40] Thornberry M P, Slebodnick C, Deck P A, Fronczek F R (2000). Organometallics.

[41] Rhode C, Lemke J, Lieb M, Metzler-Nolte N (2009). Synthesis.

[42] Nieto D, Bruña S, Montero-Campillo M M, Perles J, González-Vadillo A M, Méndez J, Mo O, Cuadrado I (2013). Chem Commun.

[43] Sünkel K, Weigand S, Hoffmann A, Blomeyer S, Reuter C G, Vishnevskiy Y V, Mitzel N W (2015). J Am Chem Soc.

[44] Inkpen M S, Du S, Hildebrand M, White A J P, Harrison N M, Albrecht T, Long N J (2015). Organometallics.

[45] Benkeser R A, Goggin D, Schroll G (1954). J Am Chem Soc.

[46] Witte P, Lal T K, Waymouth R M (1999). Organometallics.

[47] Woodward R B, Rosenblum M, Whiting M C (1952). J Am Chem Soc.

[48] Sonoda A, Moritani I (1971). J Organomet Chem.

[49] Petrov A R, Jess K, Freytag M, Jones P G, Tamm M (2013). Organometallics.

[50] Werner G, Butenschön H (2017). Eur J Inorg Chem.

[51] Hisatome M, Tachikawa O, Sashō M, Yamakawa K (1981). J Organomet Chem.

[52] Kasahara A, Izumi T, Yoshida Y, Shimizu I (1982). Bull Chem Soc Jpn.

[53] Deschenaux R, Kosztics I, Nicolet B (1995). J Mater Chem.

[54] Klett J (2018). Chem – Eur J.

[55] Bruce M I, Skelton B W, Wallis R C, Walton J K, White A H, Williams M L (1981). J Chem Soc, Chem Commun.

[56] Bruce M I, Walton J K, Williams M L, Patrick J M, Skelton B W, White A H (1983). J Chem Soc, Dalton Trans.

[57] Rausch M D, Ciappenelli D J (1967). J Organomet Chem.

[58] Sanders R, Mueller-Westerhoff U T (1996). J Organomet Chem.

[59] Bishop J J, Davison A, Katcher M L, Lichtenberg D W, Merrill R E, Smart J C (1971). J Organomet Chem.

[60] Butler I R, Cullen W R, Ni J, Rettig S J (1985). Organometallics.

[61] Reeves P C (1977). Org Synth.

[62] Kaim W, Klein A (2008). Spectroelectrochemistry.

[63] Kaim W, Fiedler J (2009). Chem Soc Rev.

[64] Siebler D, Linseis M, Gasi T, Carrella L M, Winter R F, Förster C, Heinze K (2011). Chem – Eur J.

[65] Neidlinger A, Ksenofontov V, Heinze K (2013). Organometallics.

[66] Kienz T, Förster C, Heinze K (2014). Organometallics.

[67] Hüttinger K, Förster C, Heinze K (2014). Chem Commun.

[68] Preiß S, Melomedov J, Wünsche von Leupoldt A, Heinze K (2016). Chem Sci.

[69] Bertini I, Luchinat C, Parigi G (2001). Solution NMR of paramagnetic molecules. Applications to metallobiomolecules and models. Current methods in inorganic chemistry 2.

[70] Siebler D, Förster C, Gasi T, Heinze K (2011). Organometallics.

[71] Siebler D, Förster C, Heinze K (2011). Dalton Trans.

[72] Förster C, Veit P, Ksenofontov V, Heinze K (2015). Chem Commun.

[73] Preiß S, Förster C, Otto S, Bauer M, Müller P, Hinderberger D, Haeri H H, Carella L, Heinze K (2017). Nat Chem.

[74] Gubin S P (1970). Pure Appl Chem.

[75] Hansch C, Leo A, Taft R W (1991). Chem Rev.

[76] Beck, W, Woisetschläger Ο E, Mayer P (2001). Z Kristallogr - New Cryst Struct.

[77] Cetina M, Jukić M, Rapic V, Golobic A (2003). Acta Crystallogr, Sect C: Struct Chem.

[R78] 78For the redox couple **4**/**4+**, an additional shoulder at 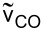 = 1710 cm^−1^ is observed during oxidation (Figure S13, Supporting Information File 1). The intensity of this shoulder also increases upon re-reduction (Figure S14, Supporting Information File 1) and hence is tentatively assigned to the C=O stretching vibration of solid **4** (Figure 3a), as a consequence of precipitation due to the lower solubility of **4** in CH_2_Cl_2_.

[79] Gray H B, Sohn Y S, Hendrickson N (1971). J Am Chem Soc.

[80] Yamaguchi Y, Ding W, Sanderson C T, Borden M L, Morgan M J, Kutal C (2007). Coord Chem Rev.

[81] Sohn Y S, Hendrickson D N, Gray H B (1970). J Am Chem Soc.

[82] Hendrickson D N, Sohn Y S, Duggan D M, Gray H B (1973). J Chem Phys.

[83] Fulara J, Filipkowski K, Maier J P (2017). J Phys Chem C.

[84] Li Y L, Han L, Mei Y, Zhang J Z H (2009). Chem Phys Lett.

[85] Yáñez-S M, Moya S A, Zúñiga C, Cárdenas-Jirón G (2017). Comput Theor Chem.

[86] Salzner U (2013). J Chem Theory Comput.

[87] Bluemel J, Hebendanz N, Hudeczek P, Koehler F H, Strauss W (1992). J Am Chem Soc.

[88] Krejčik M, Daněk M, Hartl F (1991). J Electroanal Chem Interfacial Electrochem.

[89] Fulmer G R, Miller A J M, Sherden N H, Gottlieb H E, Nudelman A, Stoltz B M, Bercaw J E, Goldberg K I (2010). Organometallics.

[90] Neese F (2012). Wiley Interdiscip Rev: Comput Mol Sci.

[91] Becke A D (1993). J Chem Phys.

[92] Lee C, Yang W, Parr R G (1988). Phys Rev B.

[93] Miehlich B, Savin A, Stoll H, Preuss H (1989). Chem Phys Lett.

[94] Neese F, Wennmohs F, Hansen A, Becker U (2009). Chem Phys.

[95] Izsák R, Neese F (2011). J Chem Phys.

[96] Pantazis D A, Chen X-Y, Landis C R, Neese F (2008). J Chem Theory Comput.

[97] Sinnecker S, Rajendran A, Klamt A, Diedenhofen M, Neese F (2006). J Phys Chem A.

[98] Weigend F, Ahlrichs R (2005). Phys Chem Chem Phys.

[99] Weigend F (2006). Phys Chem Chem Phys.

