# Supplementary material for: Polysubstituted ferrocenes as tunable redox mediators
Source: Beilstein J Org Chem. 2018 May 7;14:1004–15. doi: 10.3762/bjoc.14.86 (PMC6009500; doi:10.3762/bjoc.14.86)
Supplement: File 1 — Mediators measured and calculated spectra, IR data and Cartesian coordinates. [file Beilstein_J_Org_Chem-14-1004-s001.pdf]

Supporting Information

for

Polysubstituted ferrocenes as tunable redox mediators

Sven D. Waniek[1], Jan Klett*[1], Christoph Förster*[1] and Katja Heinze*[1]

Address: [1]Institute of Inorganic Chemistry and Analytical Chemistry, Johannes Gutenberg University Mainz, Duesbergweg 10–14, D-55128 Mainz, Germany

Email: Jan Klett - klettj@uni-mainz.de; Christoph Förster - cfoerster@uni-mainz.de; Katja Heinze - katja.heinze@uni-mainz.de

* Corresponding author

Measured and calculated spectra, IR data and Cartesian coordinates

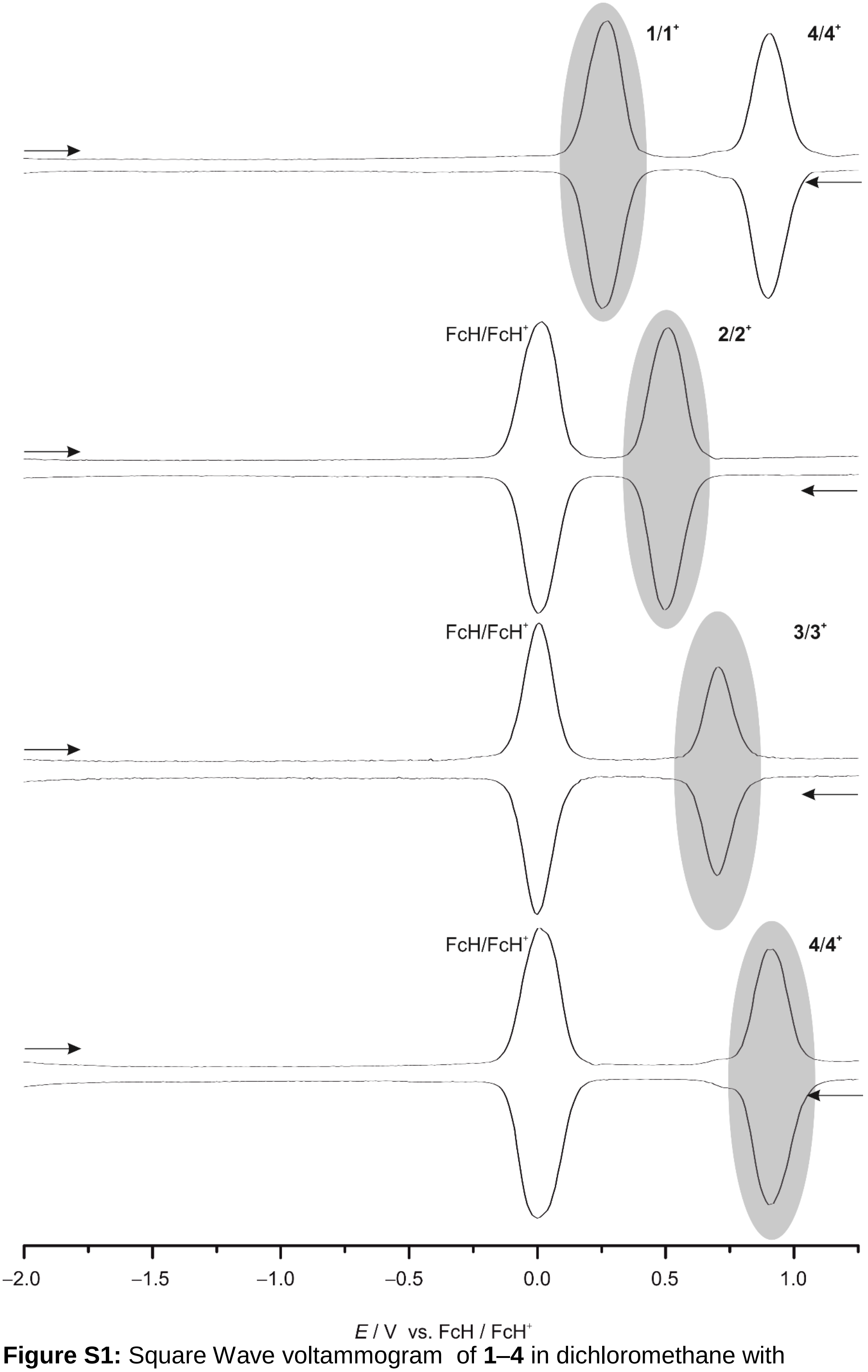

Figure S1: Square Wave voltammogram of **1–4** in dichloromethane with [nBu$_4$N][PF$_6$], referenced against FcH/FcH$^+$ for **2–4** and against **4/4$^+$** for the **1/1$^+$** couple.

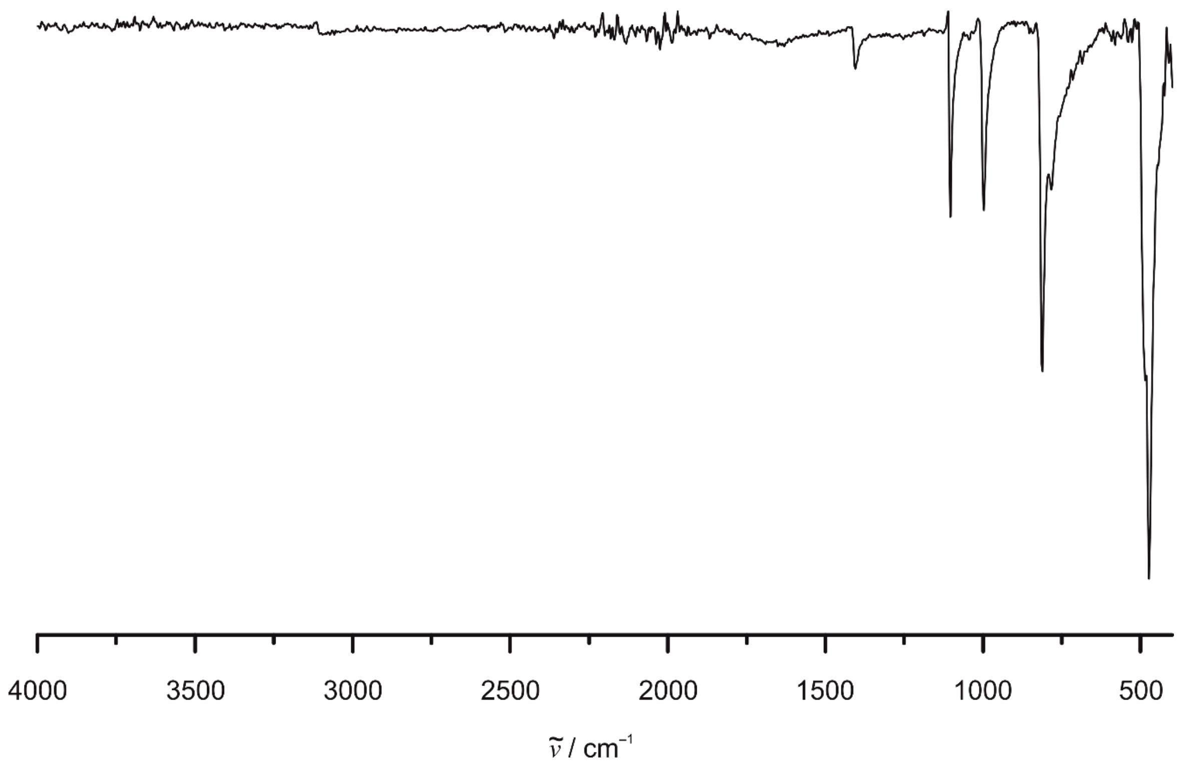

Figure S2: Solid state ATR IR spectrum of ferrocene.

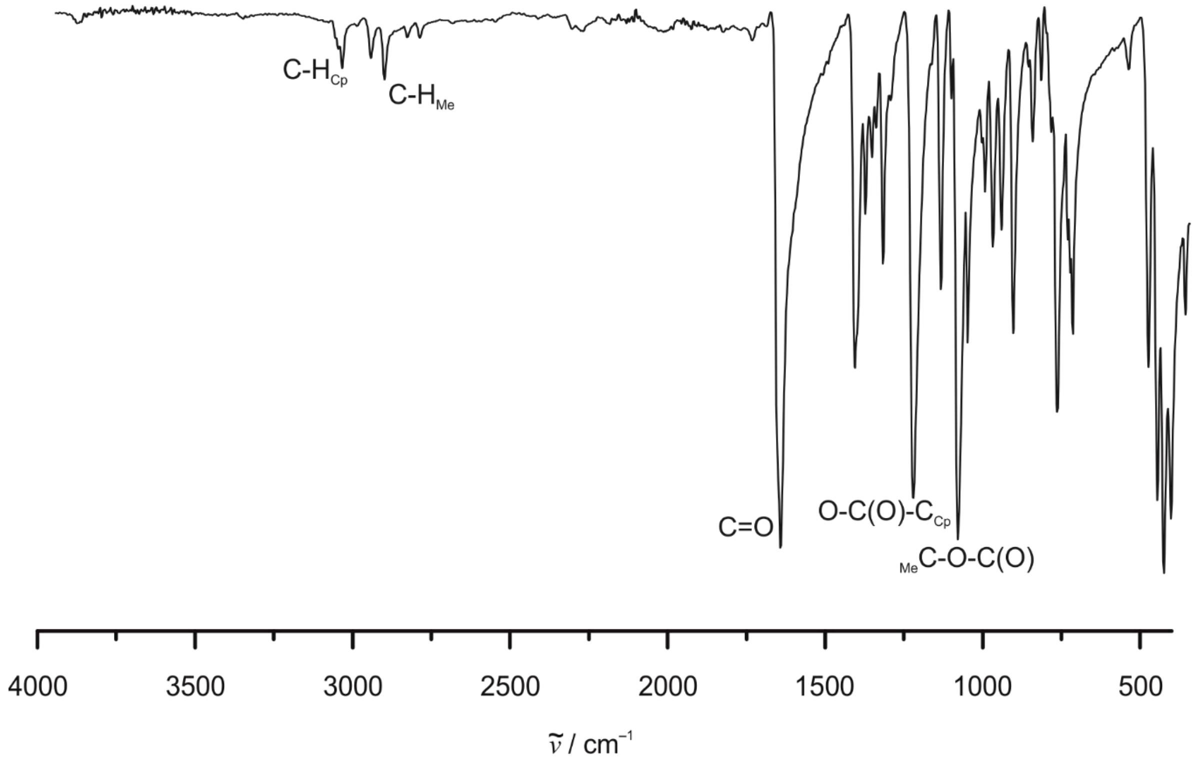

Figure S3: Solid state ATR IR spectrum of **1**.

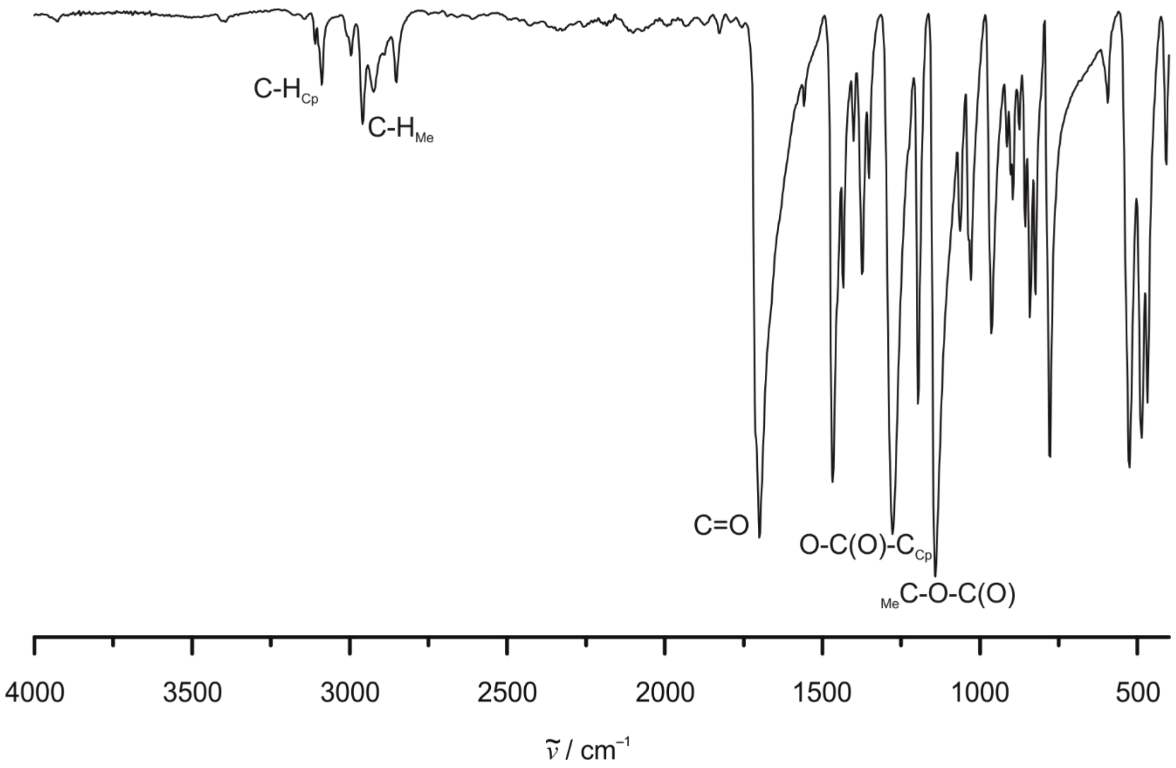

Figure S4: Solid state ATR IR spectrum of **2**.

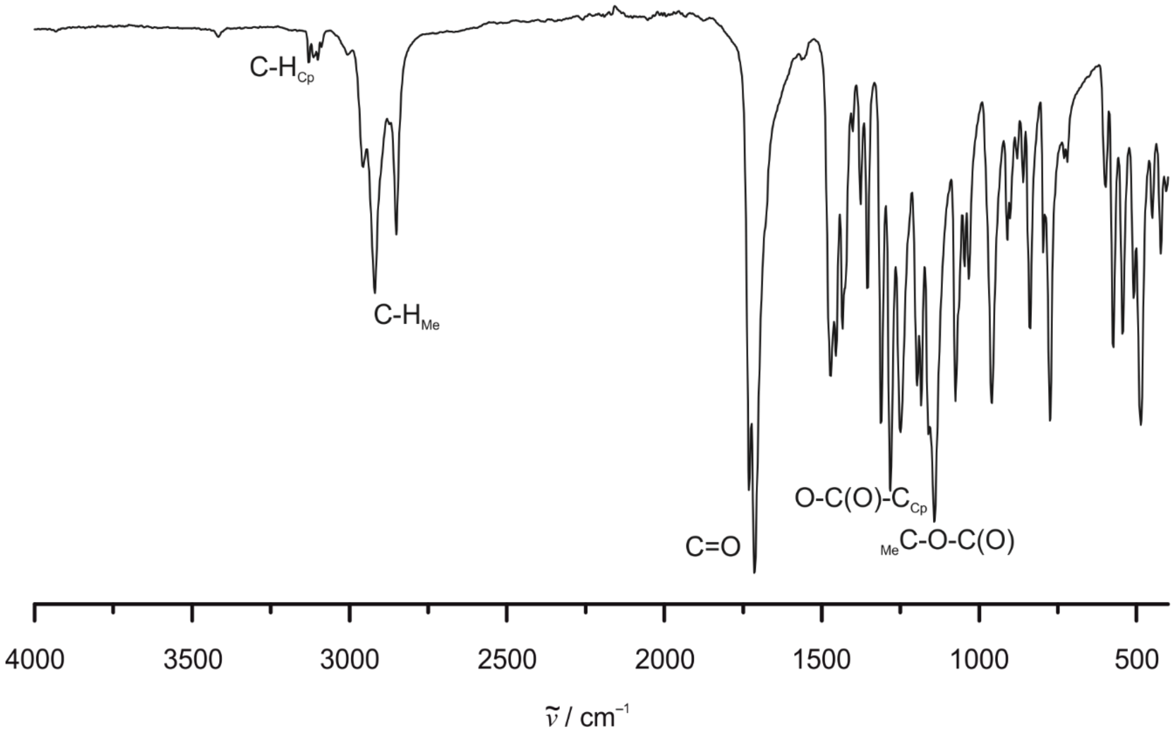

Figure S5: Solid state ATR IR spectrum of **3**.

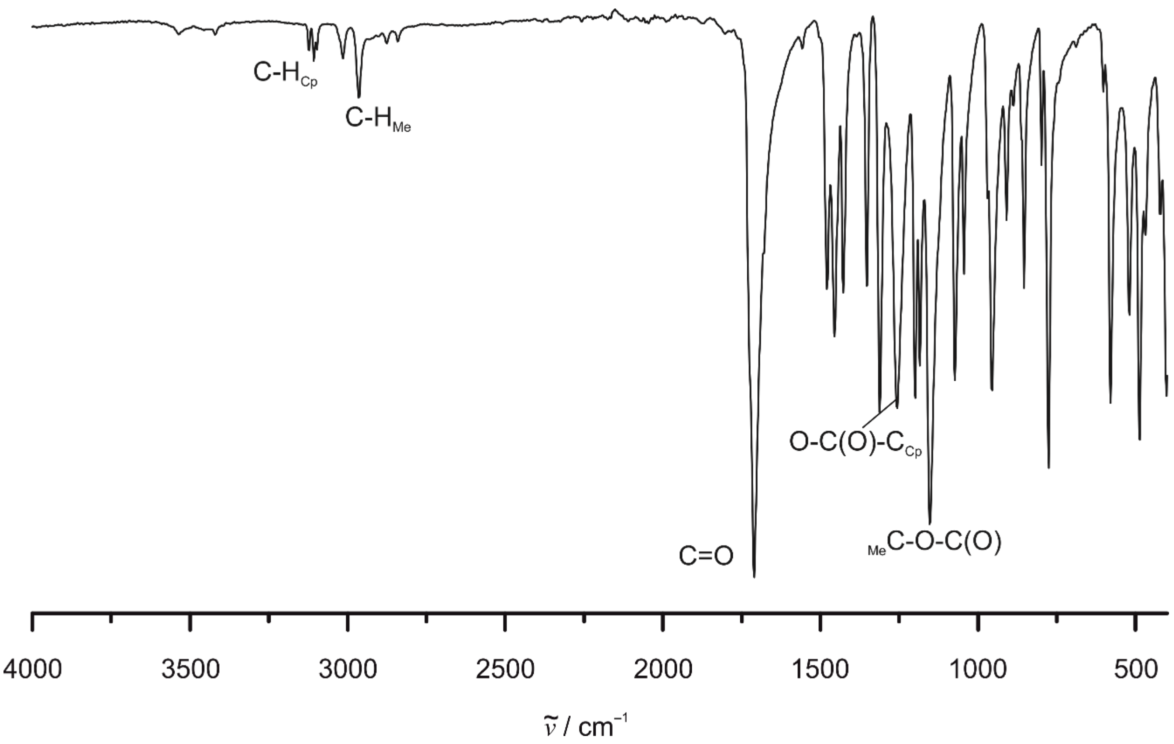

Figure S6: Solid state ATR IR-spectrum of **4**.

Table S1: IR spectroscopic data ($\tilde{\nu}_{CO}$ / cm^{-1}) of the C=O stretching vibrations of **1**–**4** and **1**$^+$–**4**$^+$, respectively and unscaled DFT[a] calculated and simulated bands ($\tilde{\nu}_{max(CO)}$ / cm^{-1}) and distinct calculated vibrations ($\tilde{\nu}_{CO}$ / cm^{-1}).

	**solid**	**solution**[b]	**DFT** $\tilde{\nu}_{max(CO)}$ ($\tilde{\nu}_{CO}$)
**1**	1709 (sh[c]), 1699	1712	1710
**2**	1709 (sh), 1699	1716	1712 (1715, 1711)
**3**	1730, 1712, 1678 (sh)	1720	1721 (1727, 1720, 1717)
**4**	1724 (sh), 1709, 1678 (sh)	1724	1724 (1733, 1724, 1722, 1720)
**1**$^+$	–	1738	1735
**2**$^+$	–	1740	1743 (1743, 1740)
**3**$^+$	–	1742	1748 (1748, 1744)
**4**$^+$	–	1743	1720 (1727, 1720, 1719, 1717)

[a] B3LYP, def2-TZVP, RIJCOSX, ZORA, CPCM(CH_2Cl_2),. [b] c = 68 mM (**1**) – 2 mM (**4**), CH_2Cl_2, 0.1 M [$n$$Bu_4N$][B($C_6F_5$)$_4$]. [c] sh = shoulder.

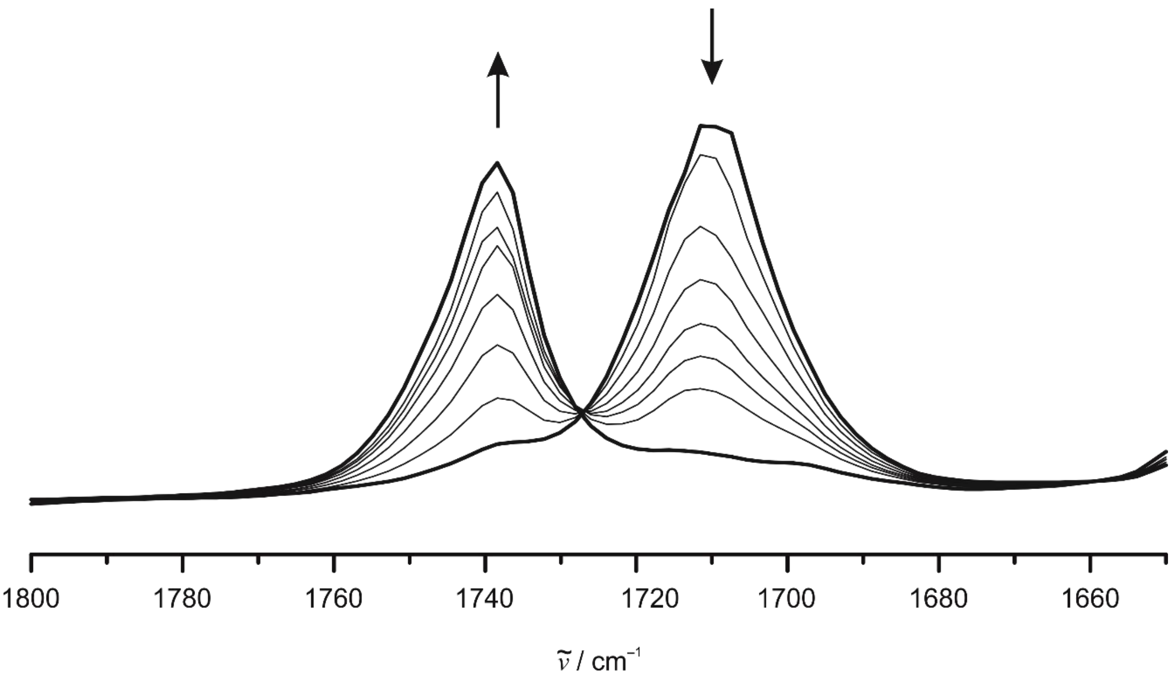

Figure S7: IR spectroelectrochemical oxidation of **1** to **1⁺** in CH_2Cl_2 / [nBu$_4$N][B(C_6F_5)$_4$] (C=O strechting vibration region, 0.3–1.0 V vs. Ag pseudo reference electrode).

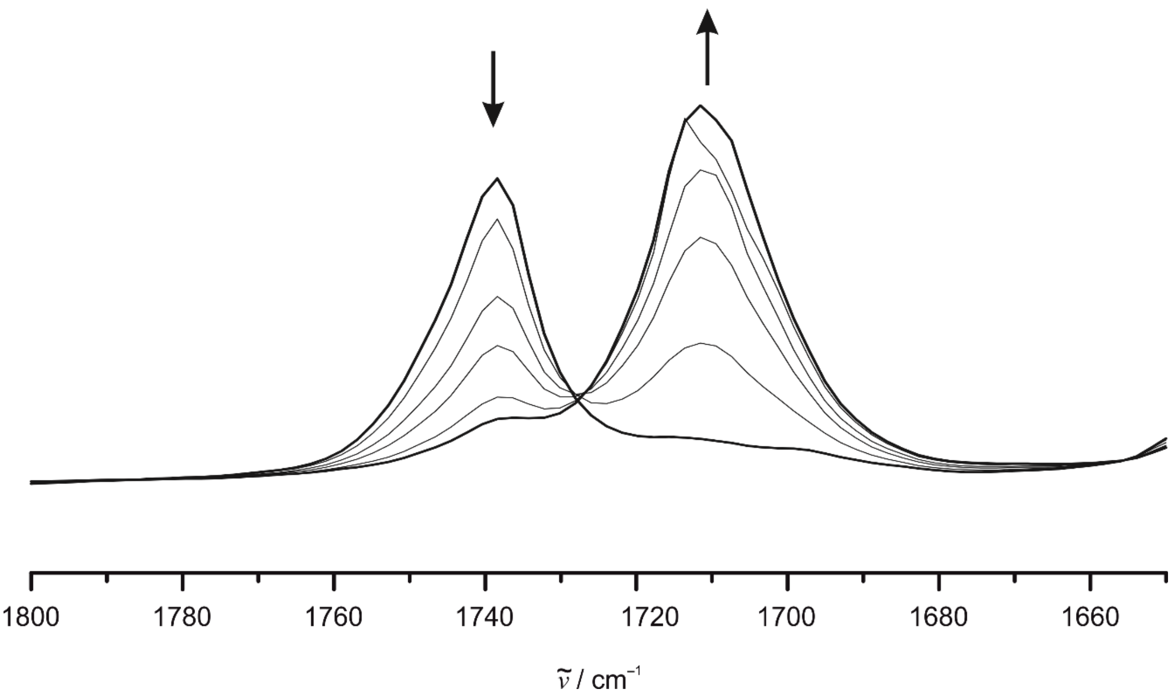

Figure S8: IR spectroelectrochemical reduction of **1⁺** to **1** in CH_2Cl_2 / [nBu$_4$N][B(C_6F_5)$_4$] (C=O strechting vibration region, 1.0–(–0.2) V vs. Ag pseudo reference electrode).

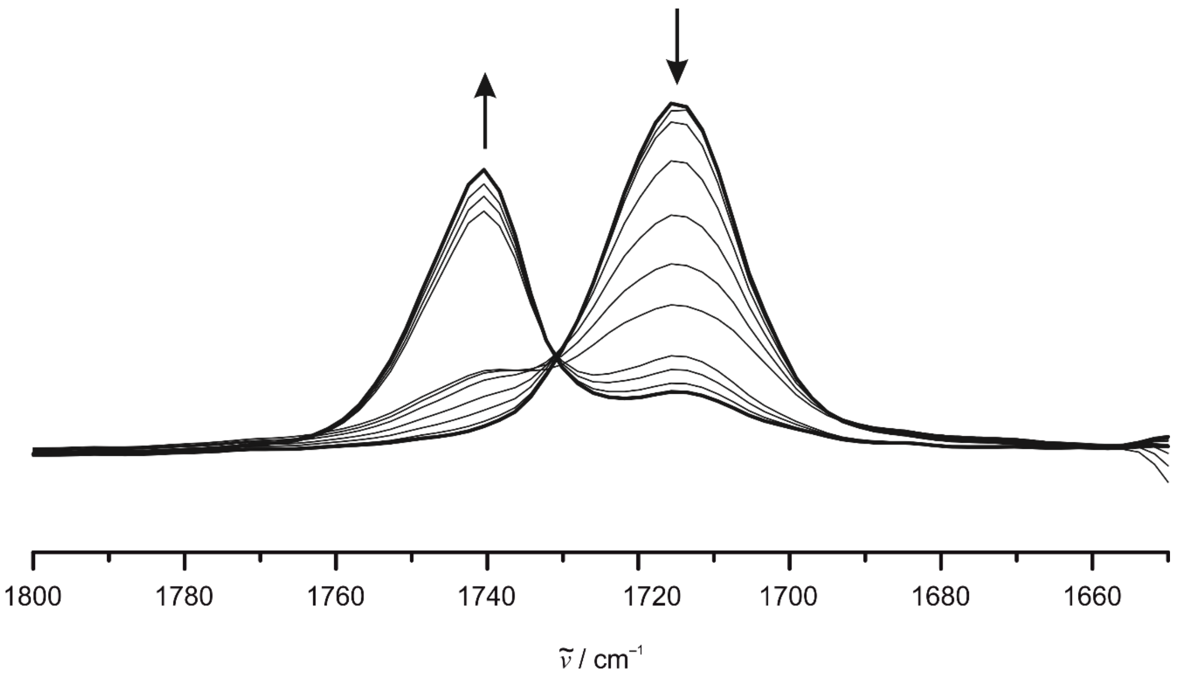

Figure S9: IR spectroelectrochemical oxidation of **2** to **2**$^+$ in CH_2Cl_2 / [nBu$_4$N][B(C_6F_5)$_4$] (C=O strechting vibration region, 0.2–1.0 V vs. Ag pseudo reference electrode).

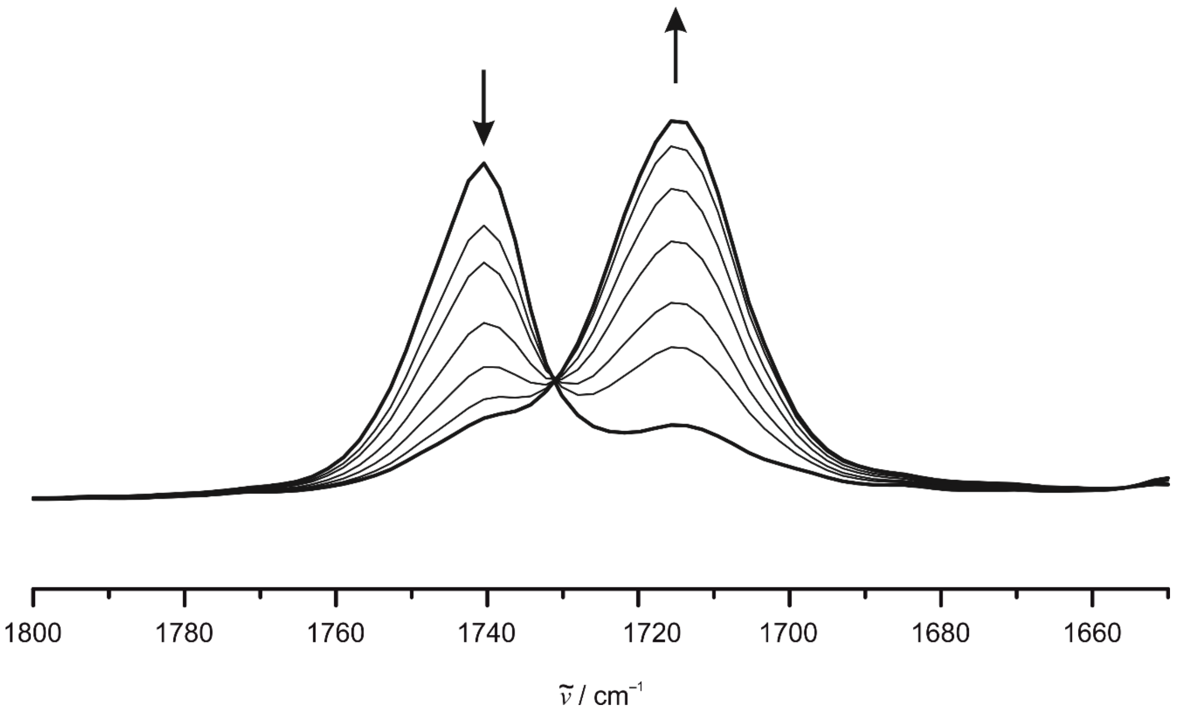

Figure S10: IR spectroelectrochemical reduction of **2**$^+$ to **2** in CH_2Cl_2 / [nBu$_4$N][B(C_6F_5)$_4$] (C=O strechting vibration region, 1.0–0.0 V vs. Ag pseudo reference electrode).

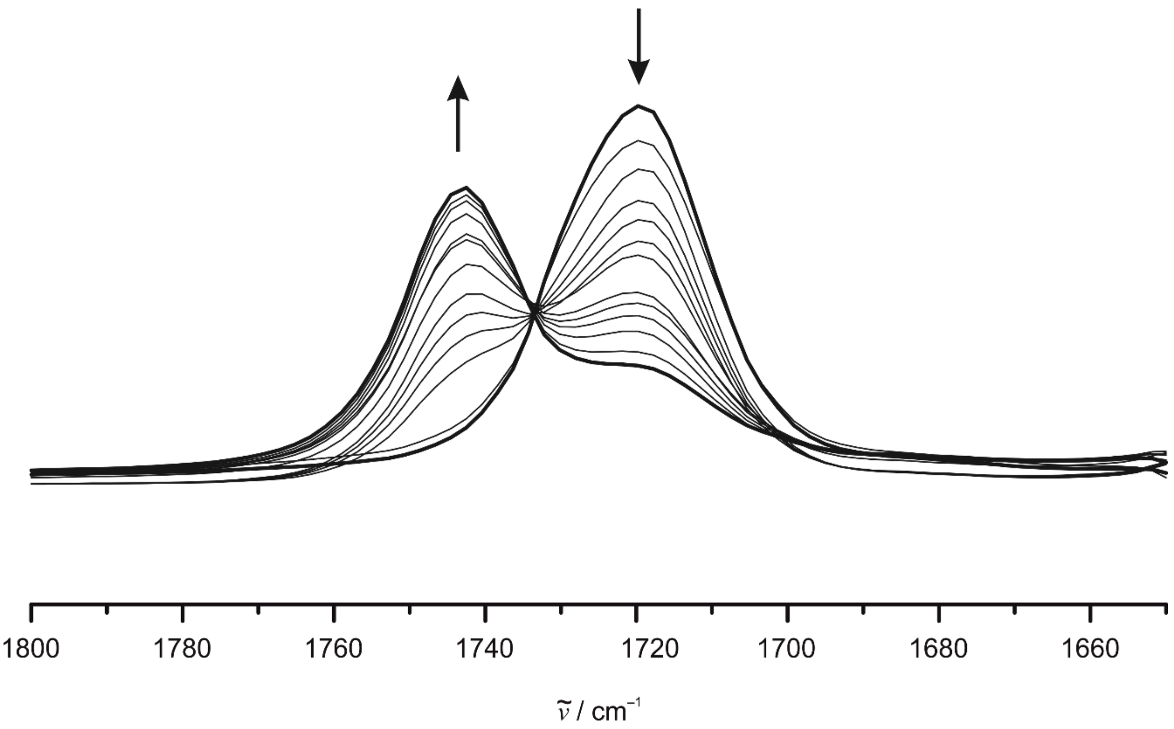

Figure S11: IR spectroelectrochemical oxidation of **3** to **3⁺** in CH_2Cl_2 / [*n*Bu_4N][$B(C_6F_5)_4$] (C=O strechting vibration region, 0.4–1.1 V vs. Ag pseudo reference electrode).

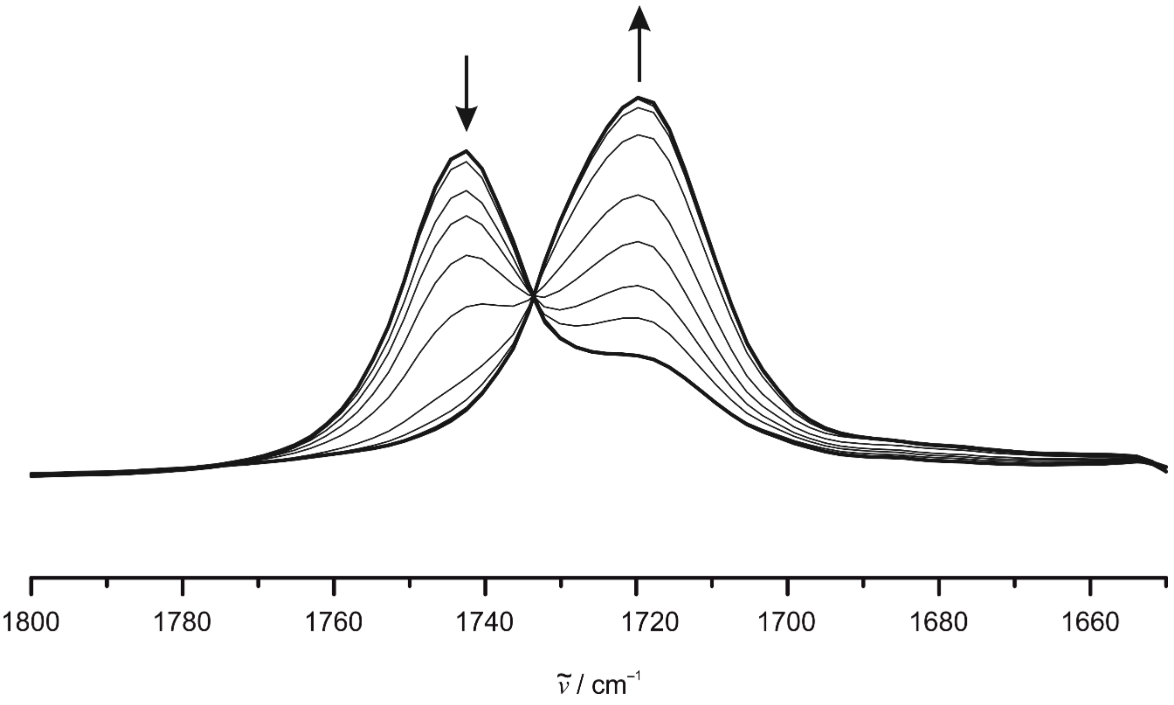

Figure S12: IR spectroelectrochemical reduction of **3⁺** to **3** in CH_2Cl_2 / [*n*Bu_4N][$B(C_6F_5)_4$] (C=O strechting vibration region, 1.1–(–0.2) V vs. Ag pseudo reference electrode).

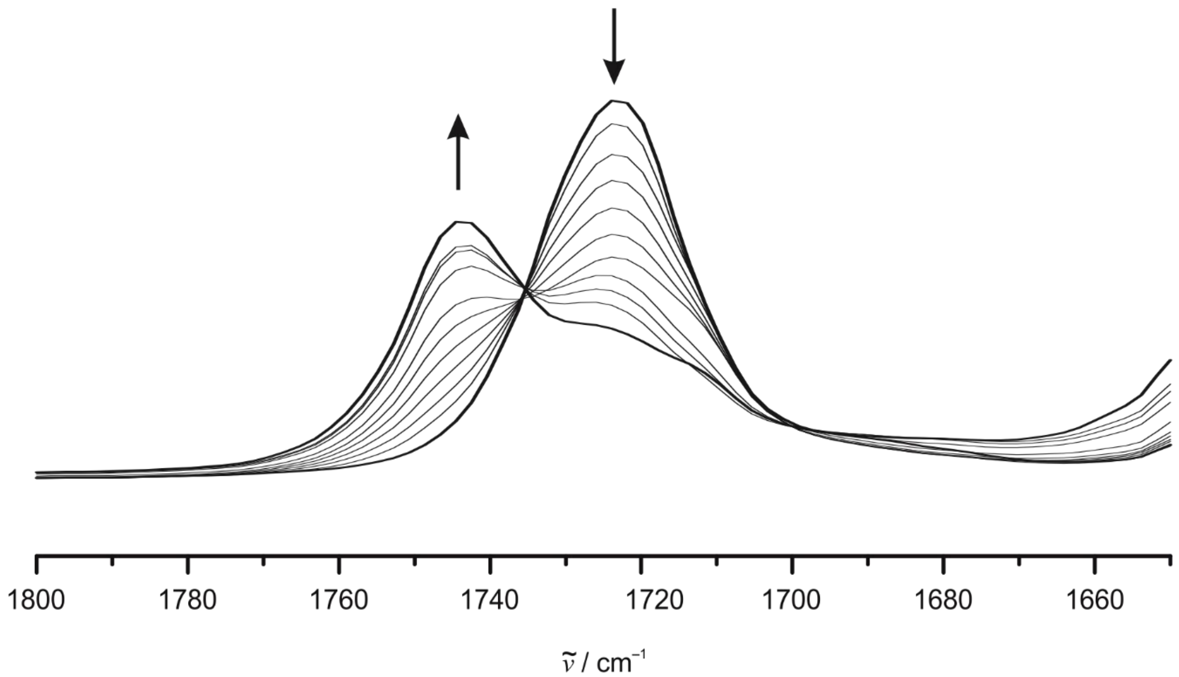

Figure S13: IR spectroelectrochemical oxidation of **4** to **4⁺** in CH_2Cl_2 / [*n*Bu_4N][B(C_6F_5)$_4$] (C=O strechting vibration region, 0.6–1.4 V vs. Ag pseudo reference electrode).

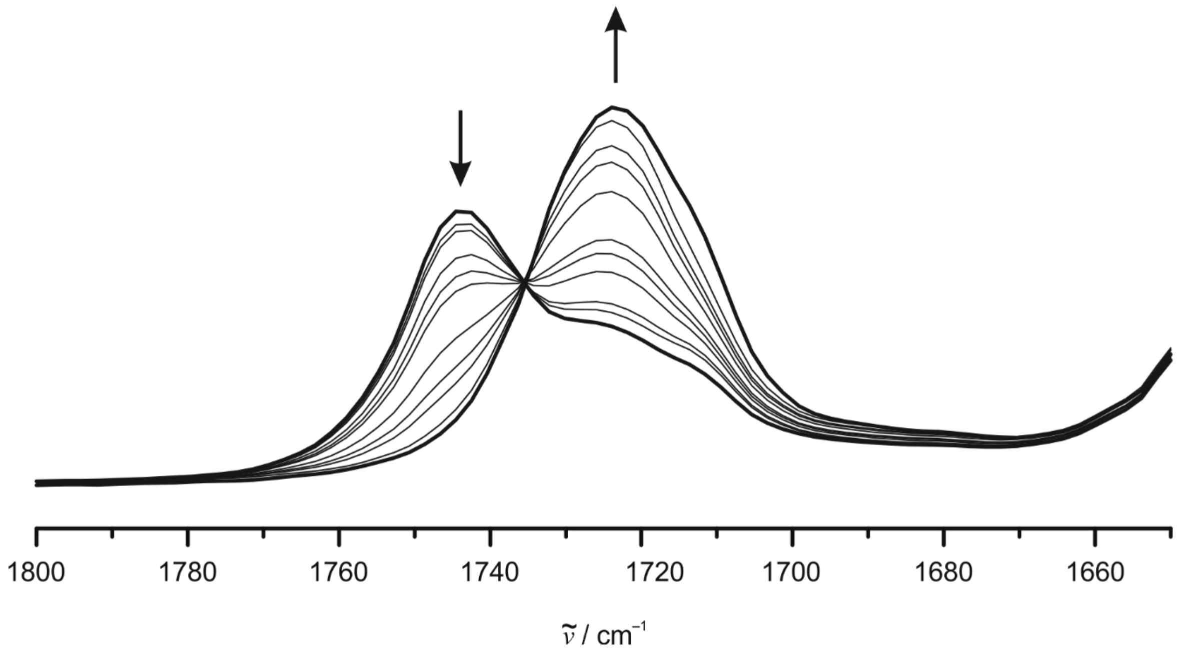

Figure S14: IR spectroelectrochemical reduction of **4⁺** to **4** in CH_2Cl_2 / [*n*Bu_4N][B(C_6F_5)$_4$] (C=O strechting vibration region, 1.4–(–0.3) V vs. Ag pseudo reference electrode).

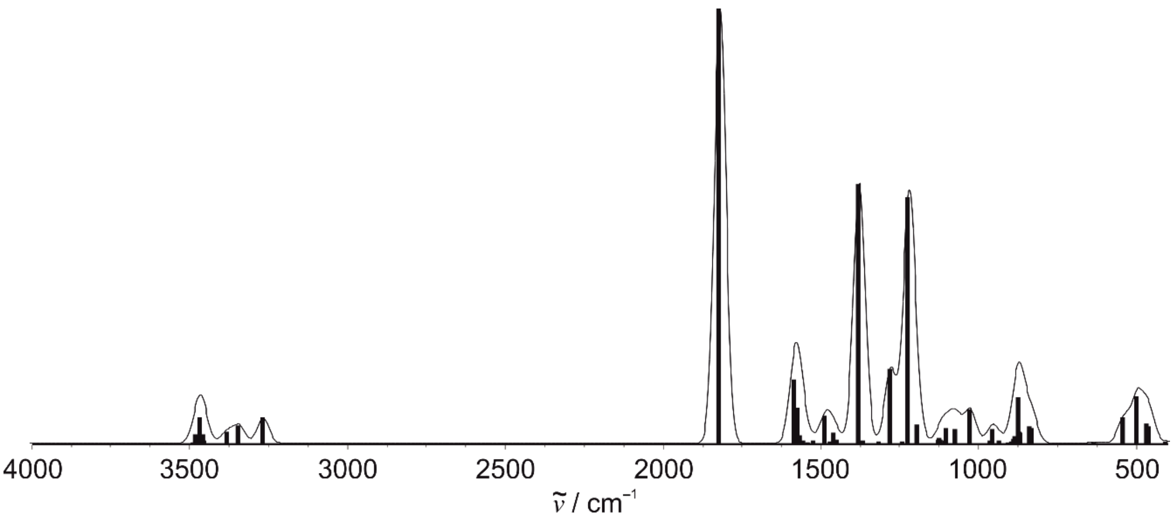

Figure S15: DFT calculated IR spectrum of **1**. [B3LYP, def2-TZVP, RIJCOSX, ZORA, CPCM(CH_2Cl_2), fwhm: 40 cm^{-1}].

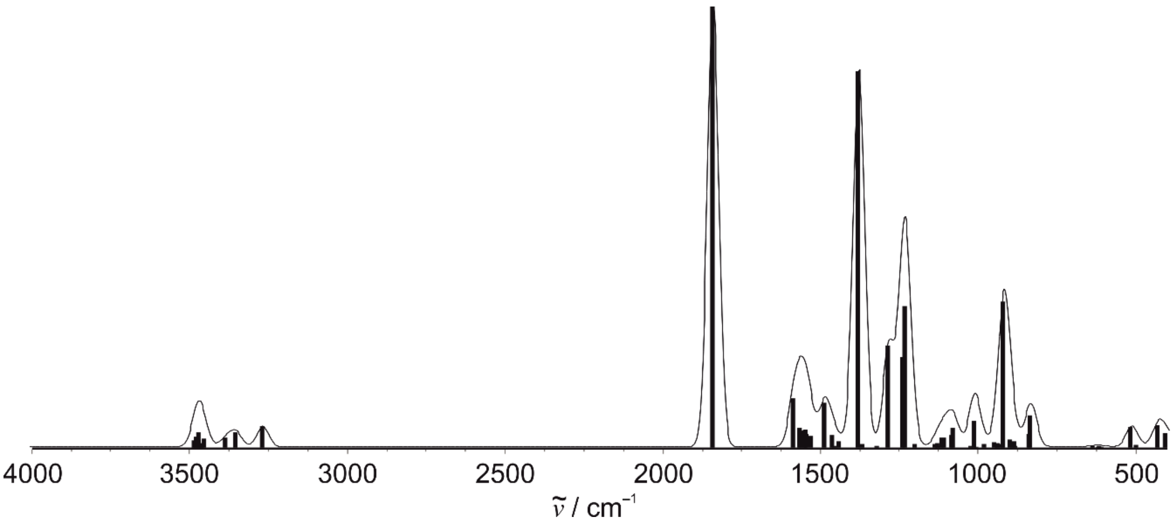

Figure S16: DFT calculated IR spectrum of **1**$^{+}$. [B3LYP, def2-TZVP, RIJCOSX, ZORA, CPCM(CH_2Cl_2), fwhm: 40 cm^{-1}].

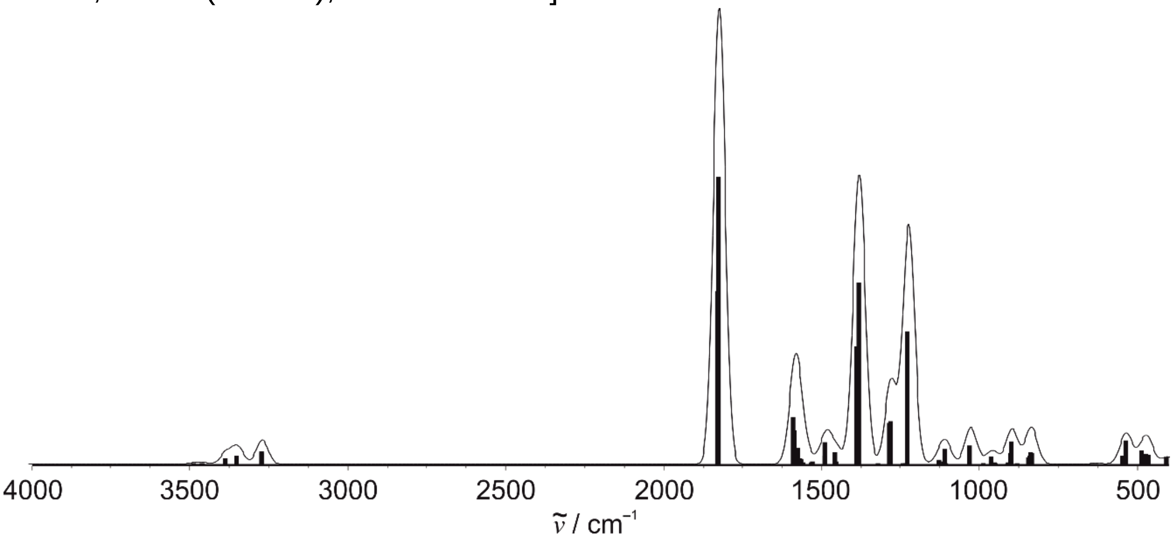

Figure S17: DFT calculated IR spectrum of **2**. [B3LYP, def2-TZVP, RIJCOSX, ZORA, CPCM(CH_2Cl_2), fwhm: 40 cm^{-1}].

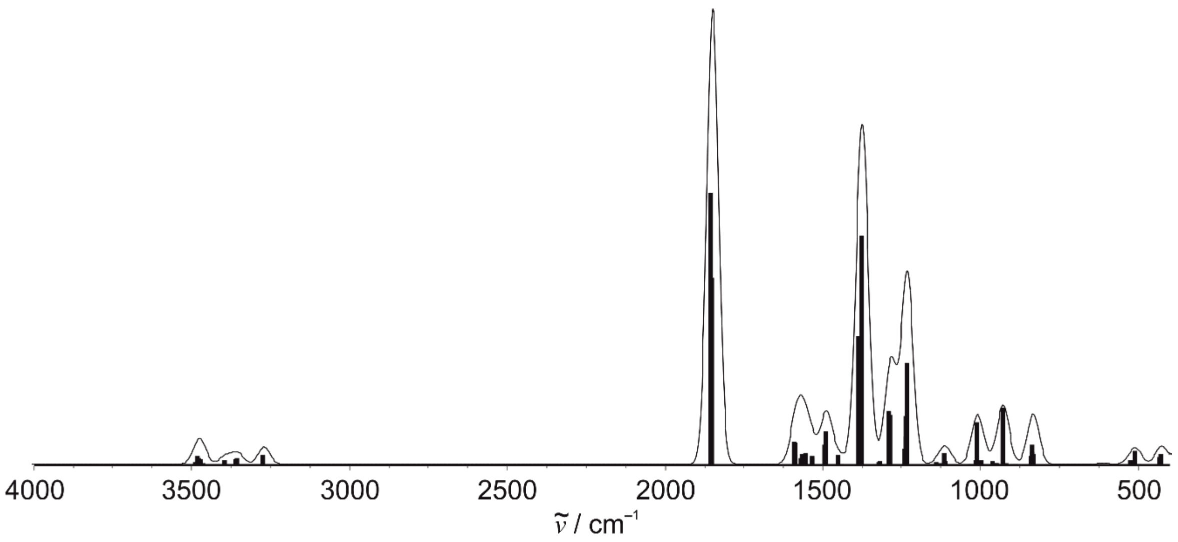

Figure S18: DFT calculated IR spectrum of **2⁺**. [B3LYP, def2-TZVP, RIJCOSX, ZORA, CPCM(CH_2Cl_2), fwhm: 40 cm^{-1}].

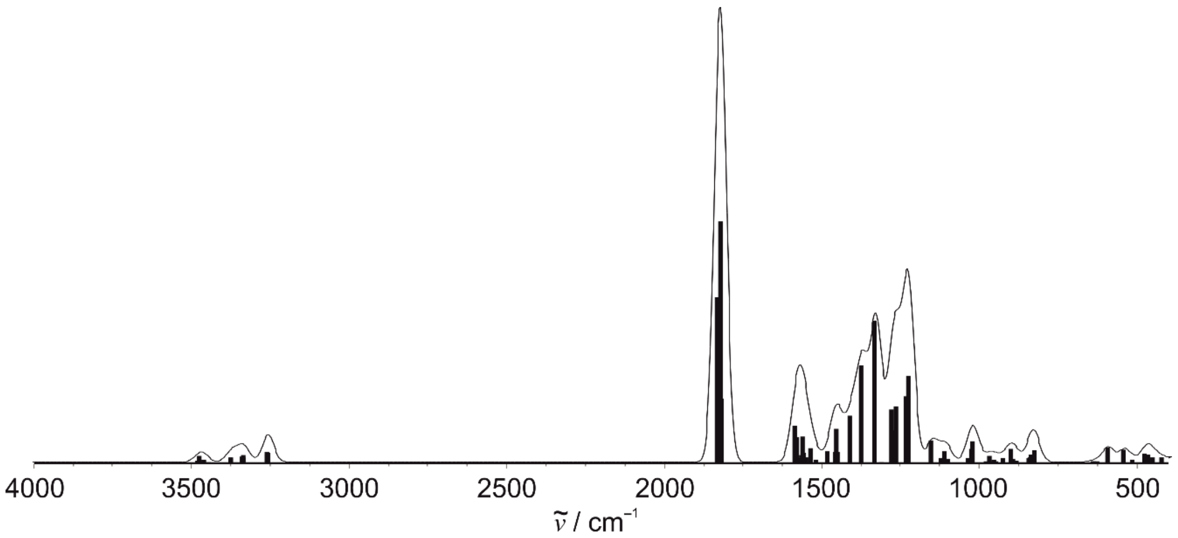

Figure S19: DFT calculated IR spectrum of **3**. [B3LYP, def2–TZVP, RIJCOSX, ZORA, CPCM(CH_2Cl_2), fwhm: 40 cm^{-1}].

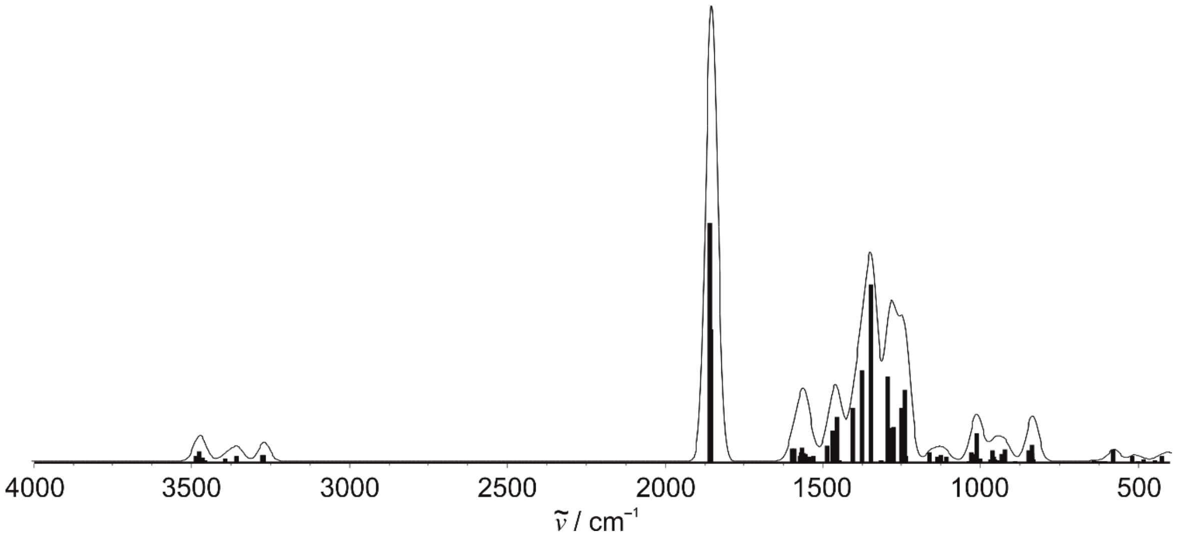

Figure S20: DFT calculated IR spectrum of **3⁺**. [B3LYP, def2-TZVP, RIJCOSX, ZORA, CPCM(CH_2Cl_2), fwhm: 40 cm^{-1}].

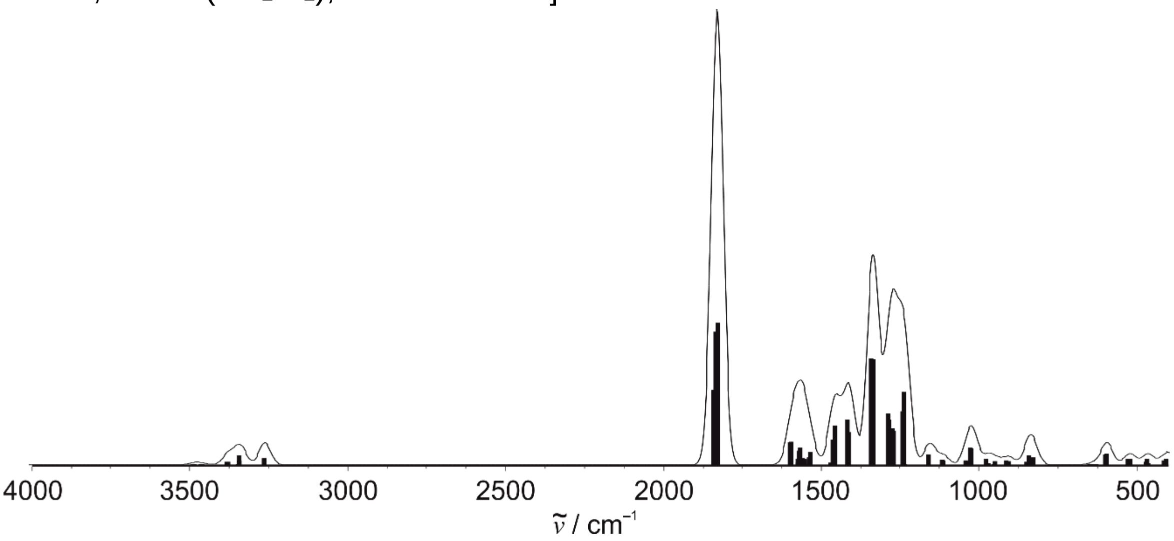

Figure S21: DFT calculated IR spectrum of **4**. [B3LYP, def2-TZVP, RIJCOSX, ZORA, CPCM(CH_2Cl_2), fwhm: 40 cm^{-1}].

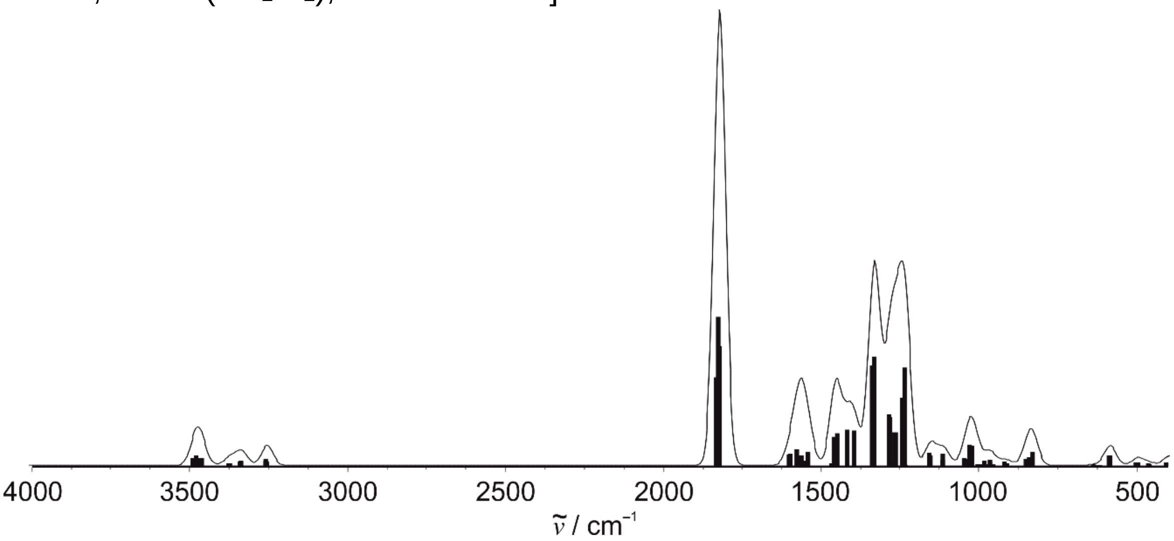

Figure S22: DFT calculated IR spectrum of **4⁺**. [B3LYP, def2-TZVP, RIJCOSX, ZORA, CPCM(CH_2Cl_2), fwhm: 40 cm^{-1}].

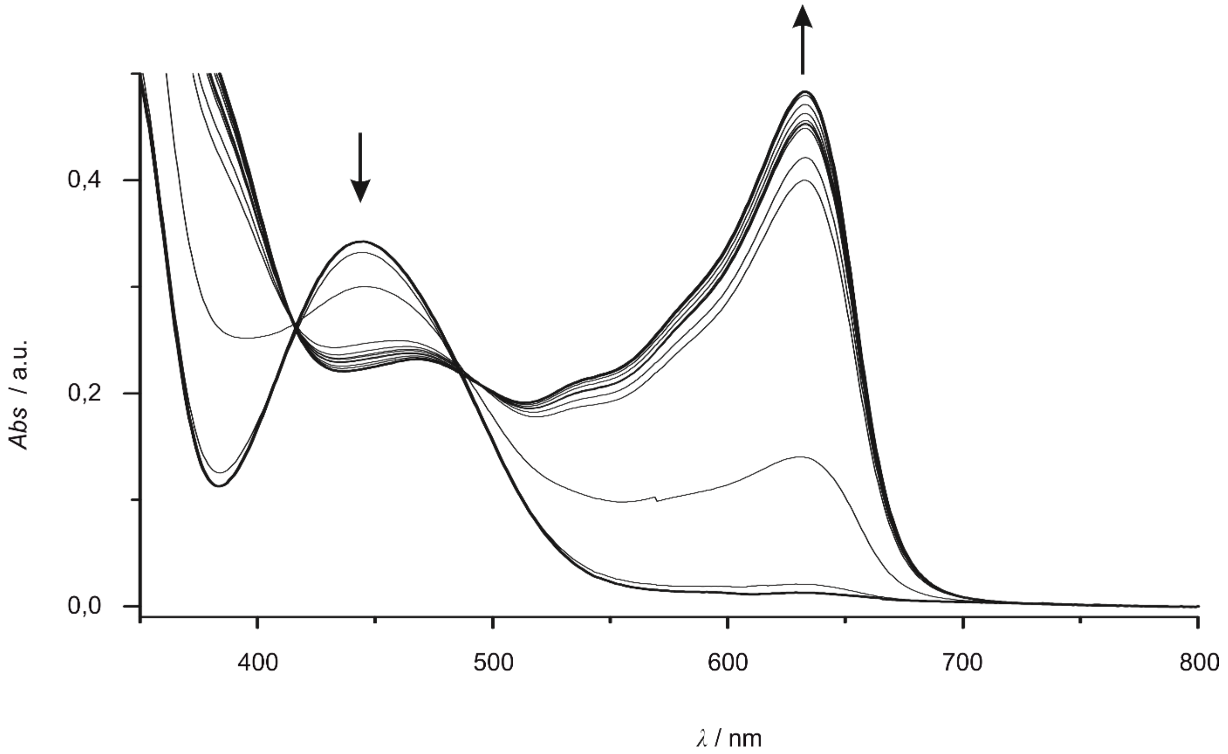

Figure S23: UV/Vis spectroelectrochemical oxidation of **1** in CH_2Cl_2 / [*n*Bu_4N][B(C_6F_5)$_4$] (0.3–1.0 V vs. Ag pseudo reference electrode).

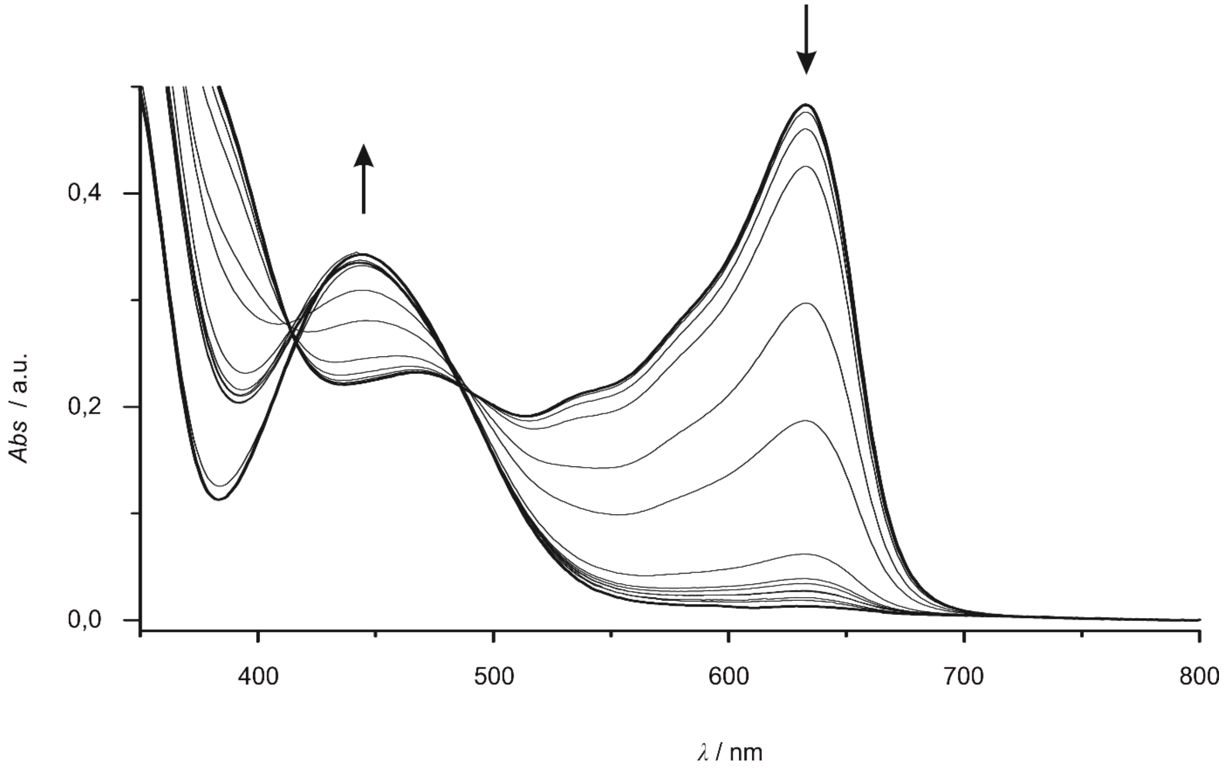

Figure S24: UV/Vis spectroelectrochemical reduction of $\mathbf{1^+}$ in CH_2Cl_2 / [*n*Bu_4N][B(C_6F_5)$_4$] (1.0–(–0.2) V vs. Ag pseudo reference electrode).

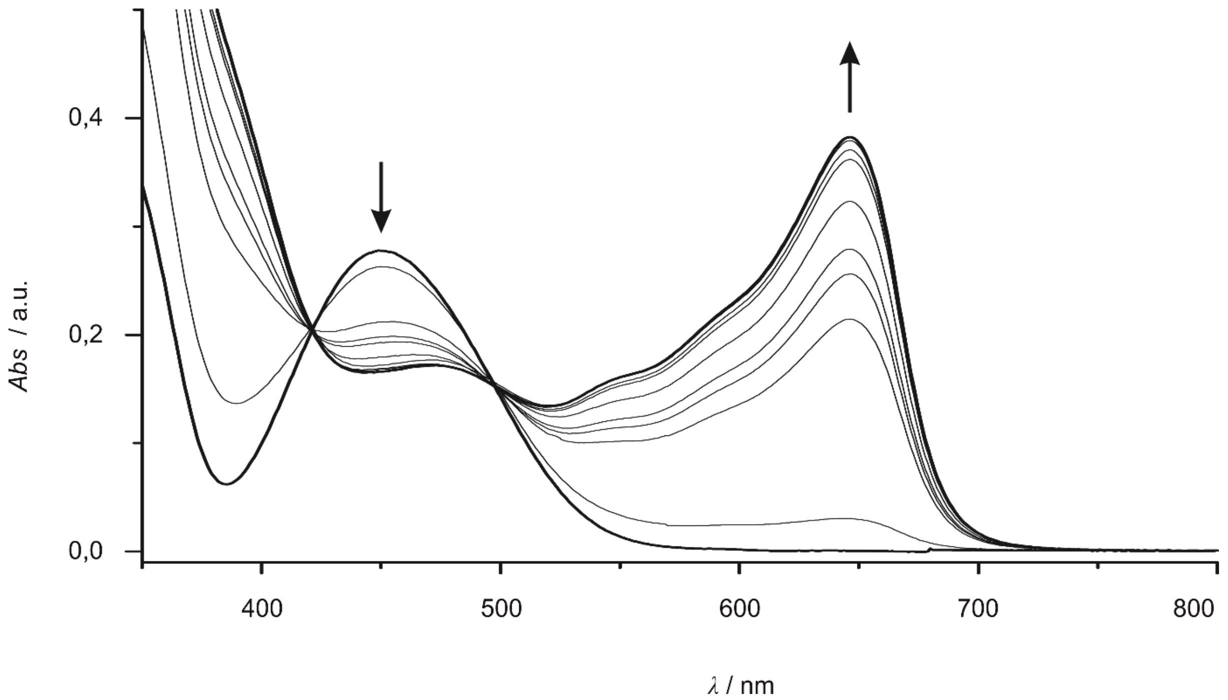

Figure S25: UV/Vis spectroelectrochemical oxidation of **2** in CH_2Cl_2 / [nBu$_4$N][B(C_6F_5)$_4$] (0.2–1.0 V vs. Ag pseudo reference electrode).

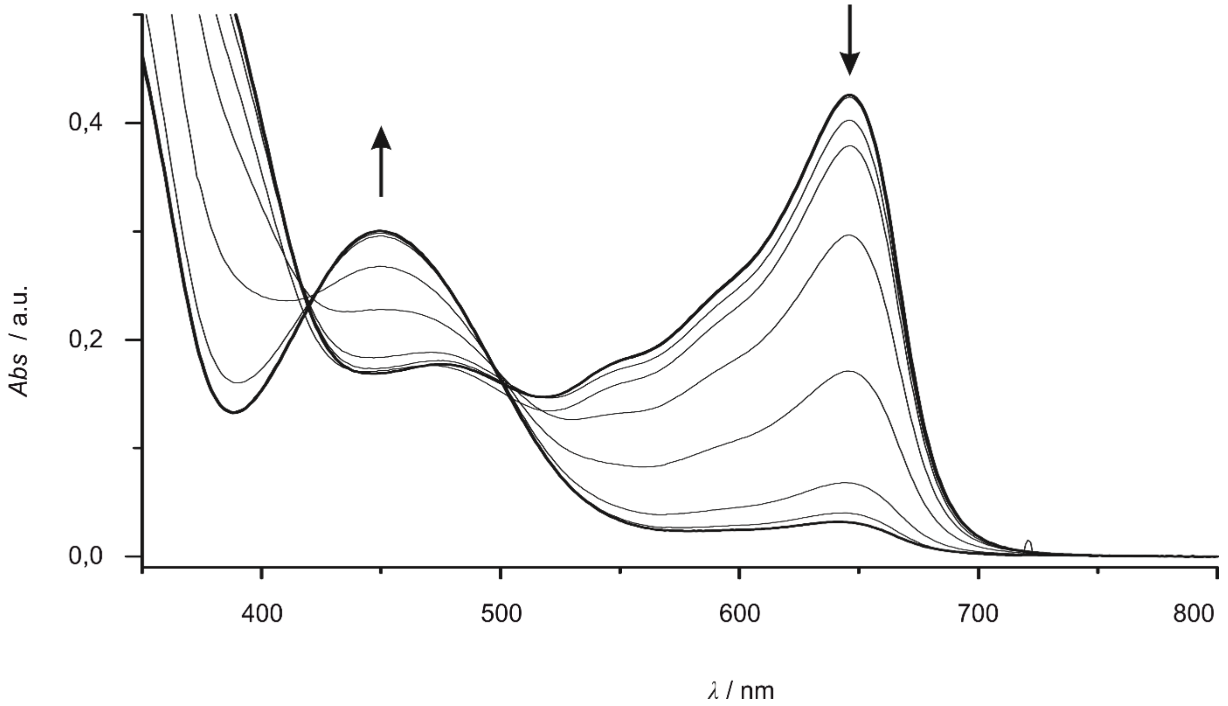

Figure S26: UV/Vis spectroelectrochemical reduction of **2**$^+$ in CH_2Cl_2 / [nBu$_4$N][B(C_6F_5)$_4$] (1.0–0.0 V vs. Ag pseudo reference electrode).

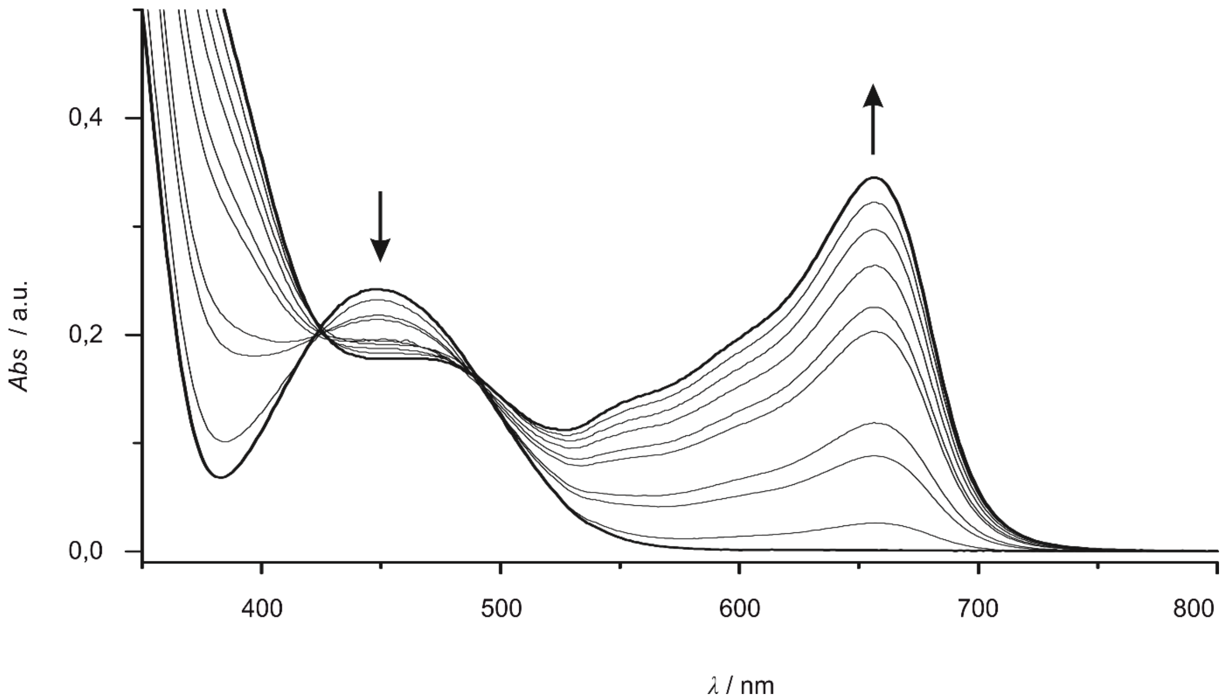

Figure S27: UV/Vis spectroelectrochemical oxidation of **3** in CH_2Cl_2 / [*n*Bu_4N][$B(C_6F_5)_4$] (0.4–1.1 V vs. Ag pseudo reference electrode).

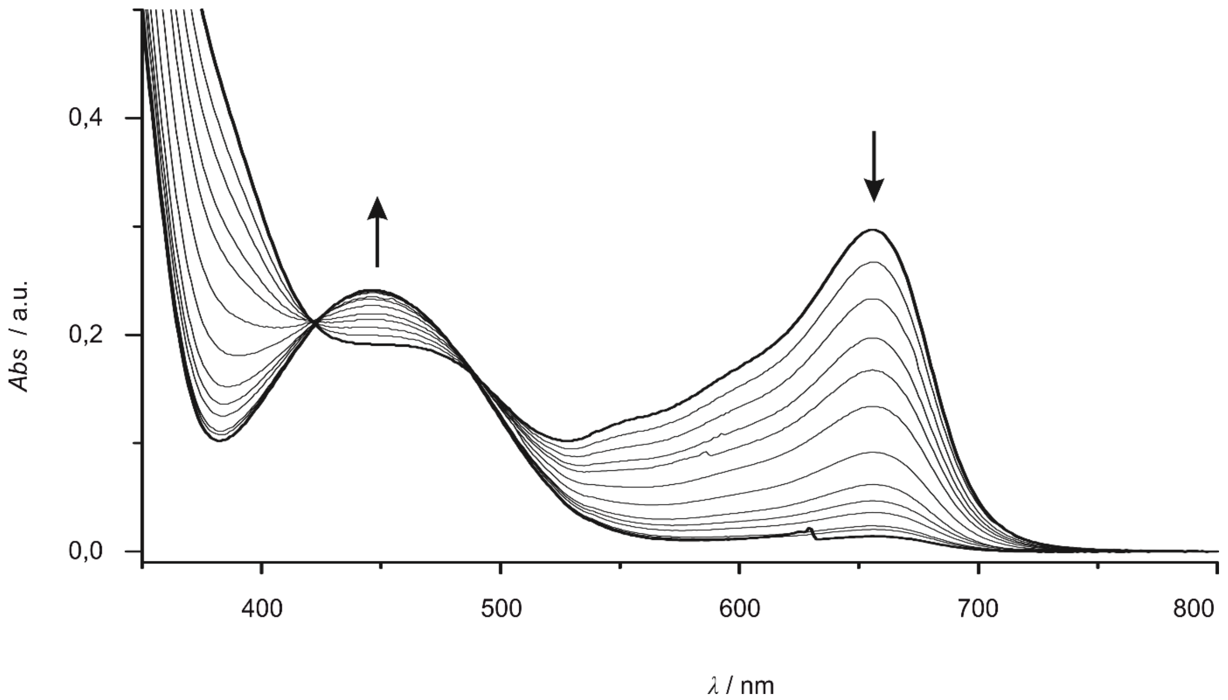

Figure S28: UV/Vis spectroelectrochemical reduction of **3**$^+$ in CH_2Cl_2 / [*n*Bu_4N][$B(C_6F_5)_4$] (1.1–(–0.2) V vs. Ag pseudo reference electrode).

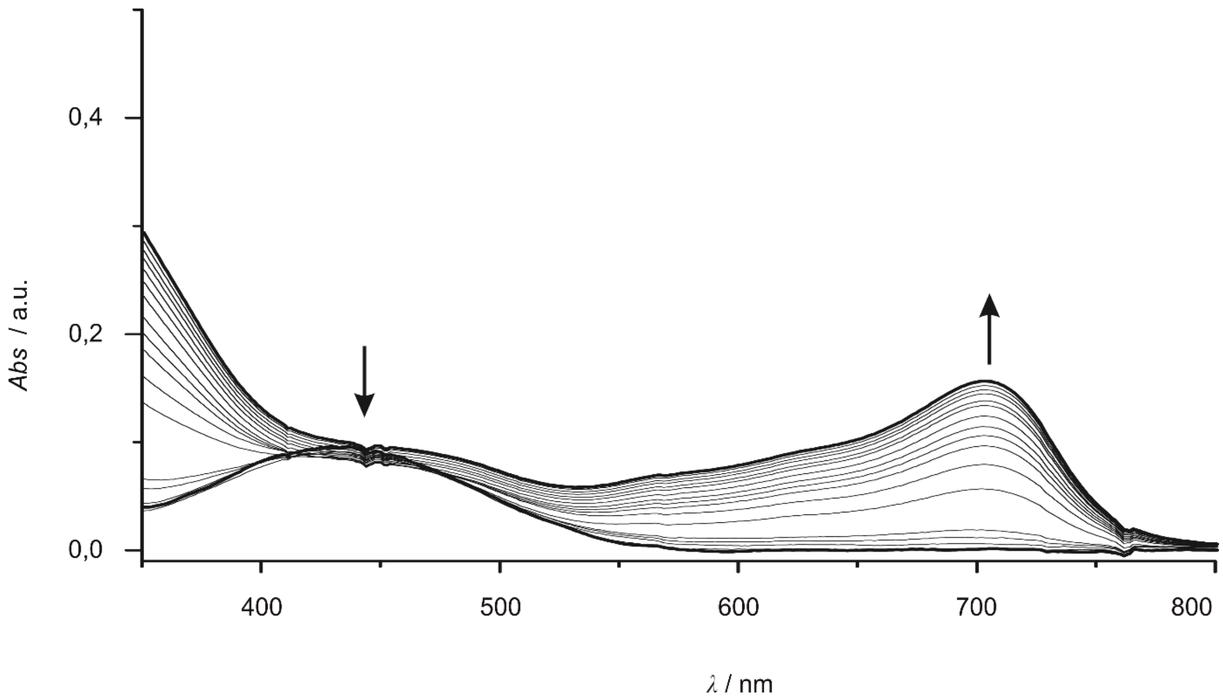

Figure S29: UV/Vis spectroelectrochemical oxidation of **4** in CH_2Cl_2 / [nBu$_4$N][B(C_6F_5)$_4$] (0.6–1.4 V vs. Ag pseudo reference electrode).

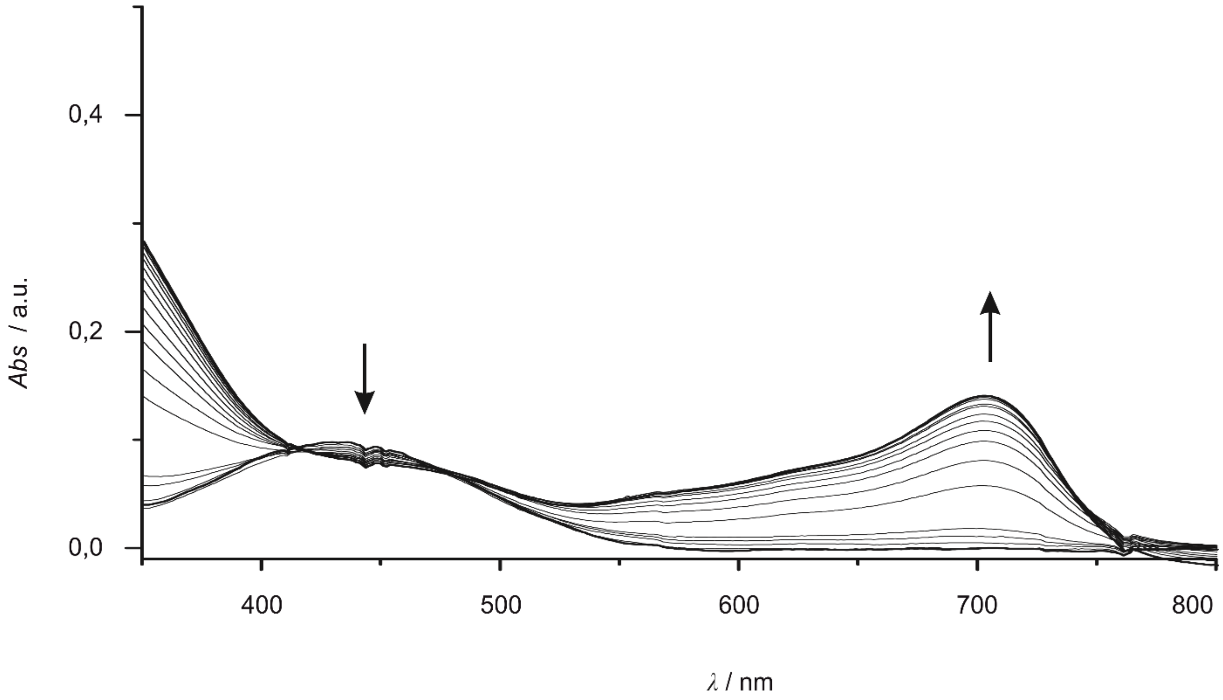

Figure S30: UV/Vis spectroelectrochemical oxidation of **4** in CH_2Cl_2 / [nBu$_4$N][B(C_6F_5)$_4$] (0.6–1.4 V vs. Ag pseudo reference electrode, with manual baseline correction).

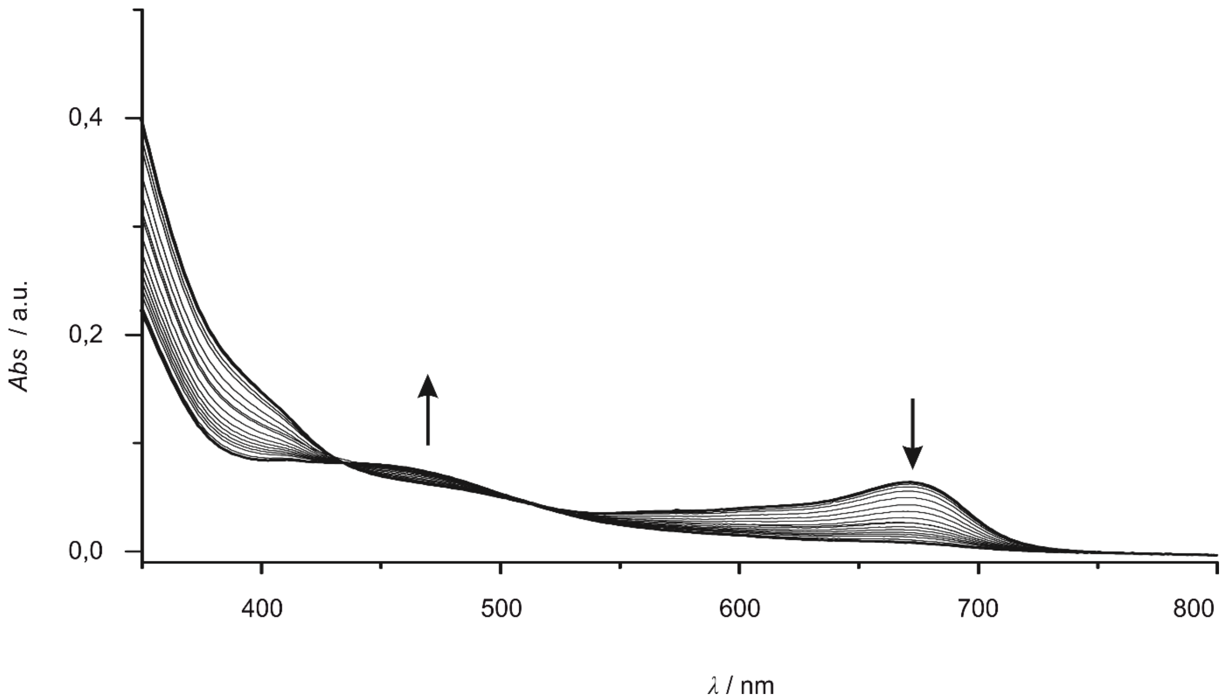

Figure S31: UV/Vis spectroelectrochemical reduction of **4⁺** in CH_2Cl_2 / [nBu$_4$N][B(C_6F_5)$_4$] (1.4–(–0.3) V vs. Ag pseudo reference electrode).

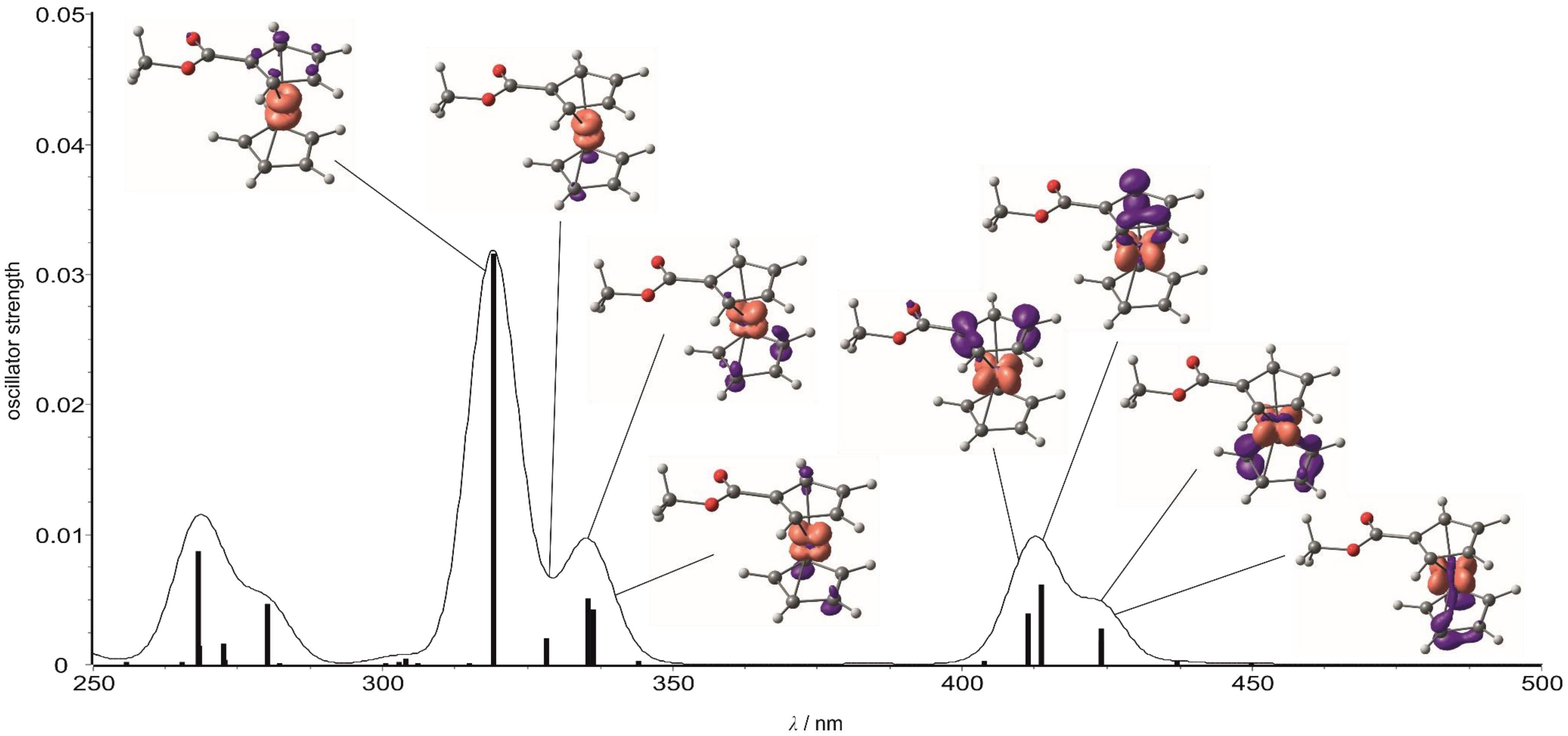

Figure S32: TD-DFT calculated UV/Vis spectrum of **1**$^{+}$ with electron difference density [B3LYP, def2-TZVP, RIJCOSX, ZORA, CPCM(CH_2Cl_2), (isosurface values 0.01 a.u.; fwhm: 10 nm)].

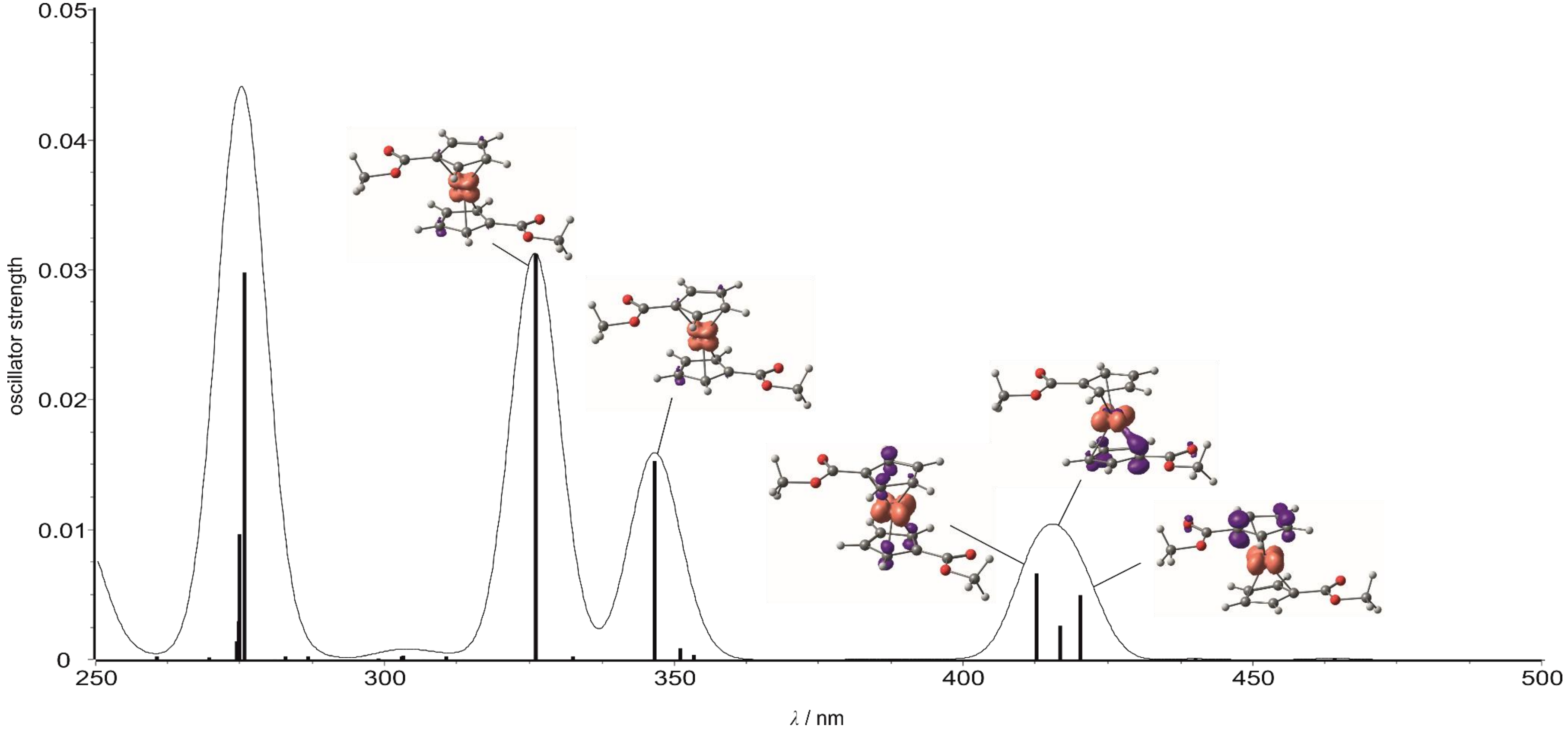

Figure S33: TD-DFT calculated UV/Vis spectrum of **2**$^{+}$ with electron difference density [B3LYP, def2-TZVP, RIJCOSX, ZORA, CPCM(CH_2Cl_2), (isosurface values 0.01 a.u.; fwhm: 10 nm)].

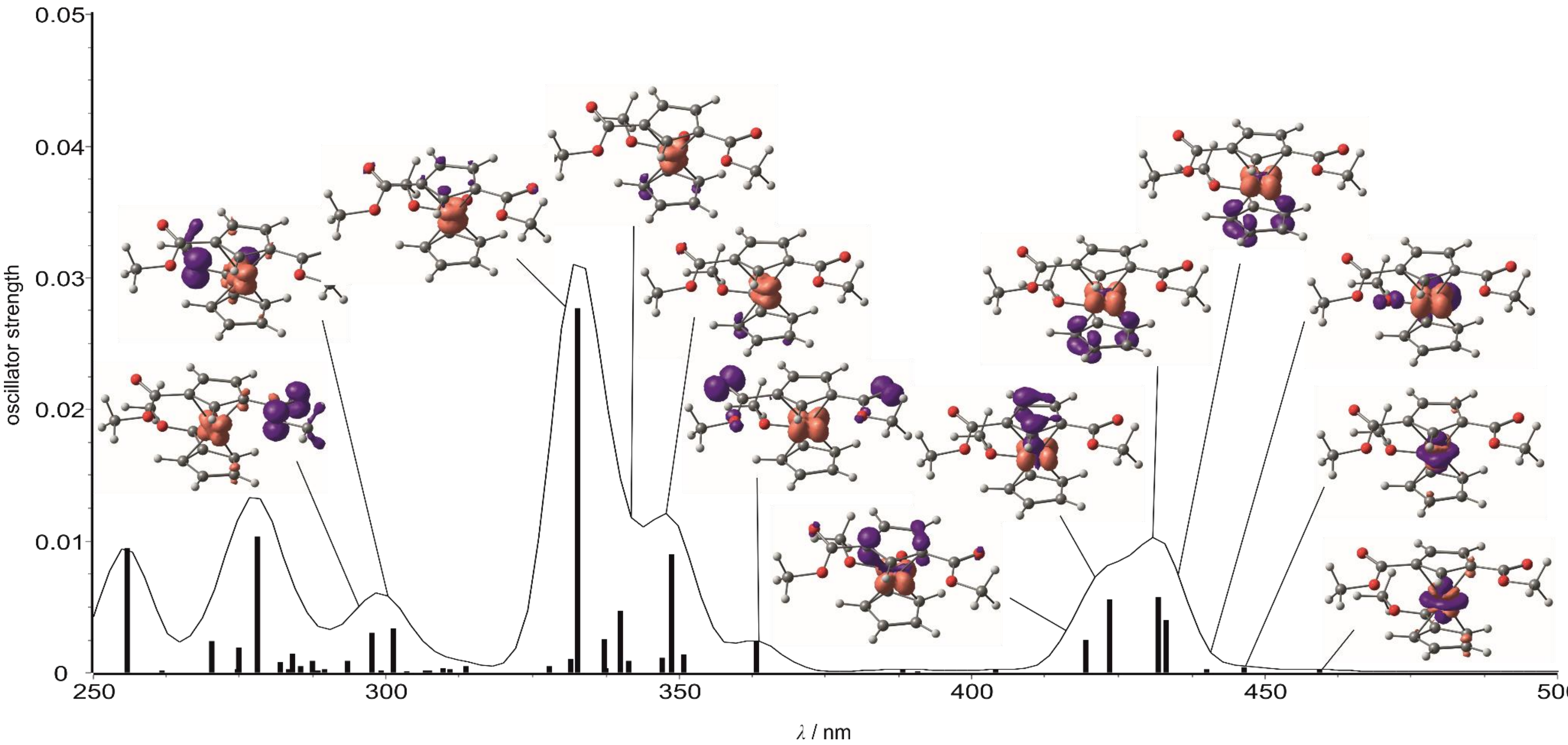

Figure S34: TD-DFT calculated UV/Vis spectrum of **3**$^{+}$ with electron difference density [B3LYP, def2-TZVP, RIJCOSX, ZORA, CPCM(CH_2Cl_2), (isosurface values 0.01 a.u.; fwhm: 10 nm)].

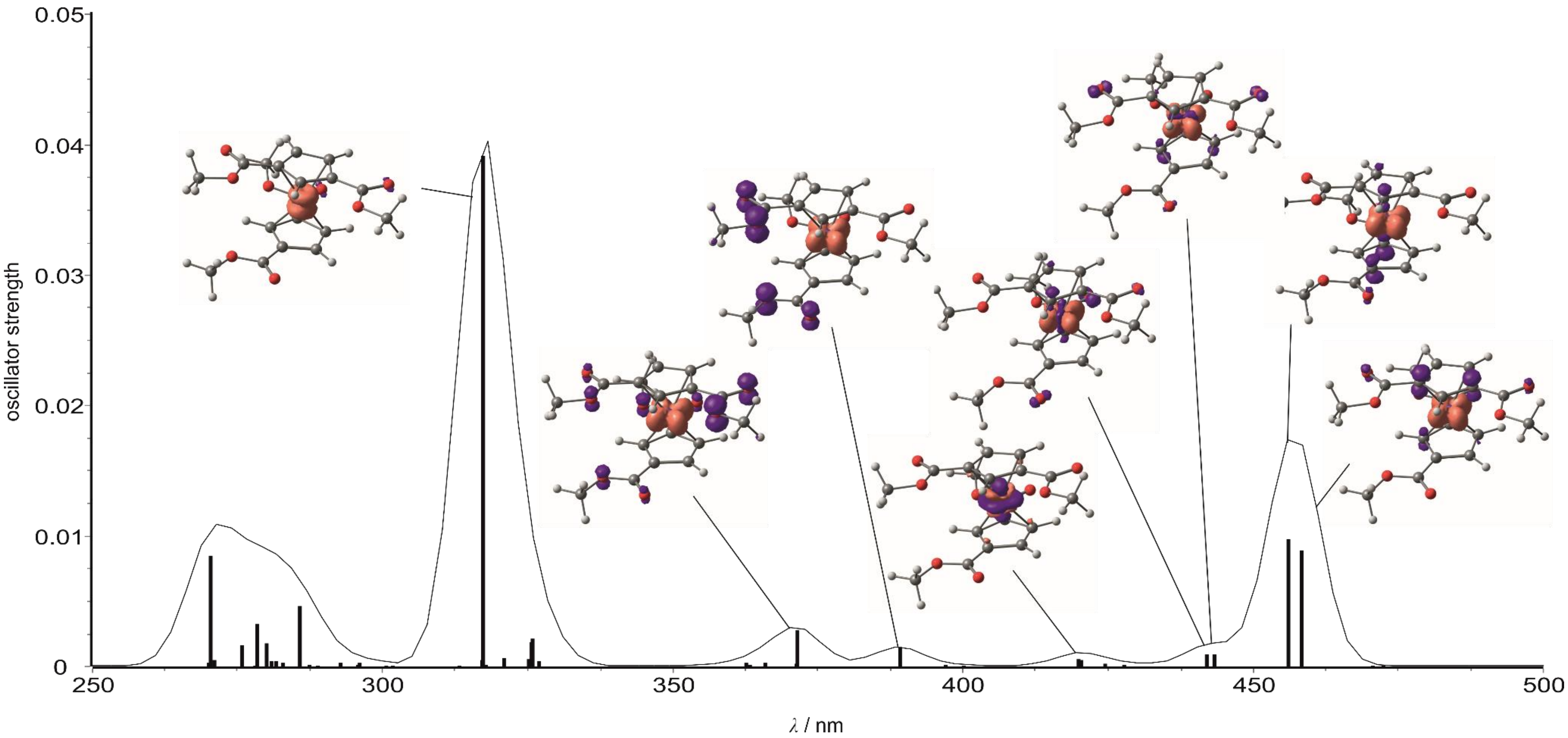

Figure S35: TD-DFT calculated UV/Vis spectrum of **4⁺** with electron difference density [B3LYP, def2-TZVP, RIJCOSX, ZORA, CPCM(CH_2Cl_2), (isosurface values 0.01 a.u.; fwhm: 10 nm)].

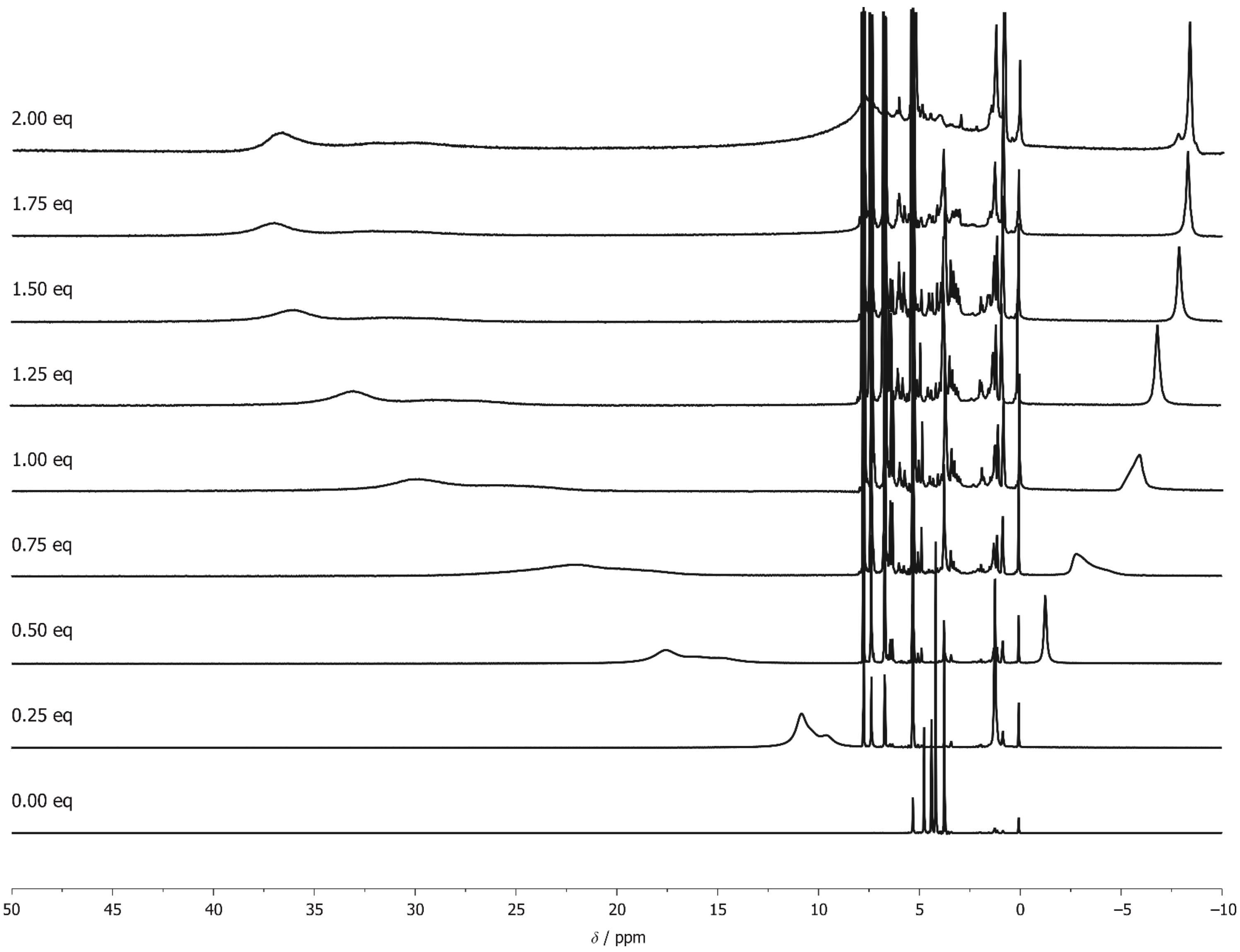

Figure S36: ^{1}H NMR oxidation titration of **1** in CD_2Cl_2 with $[N(2,4\text{-}C_6H_3Br_2)_3]^+$ as oxidant.

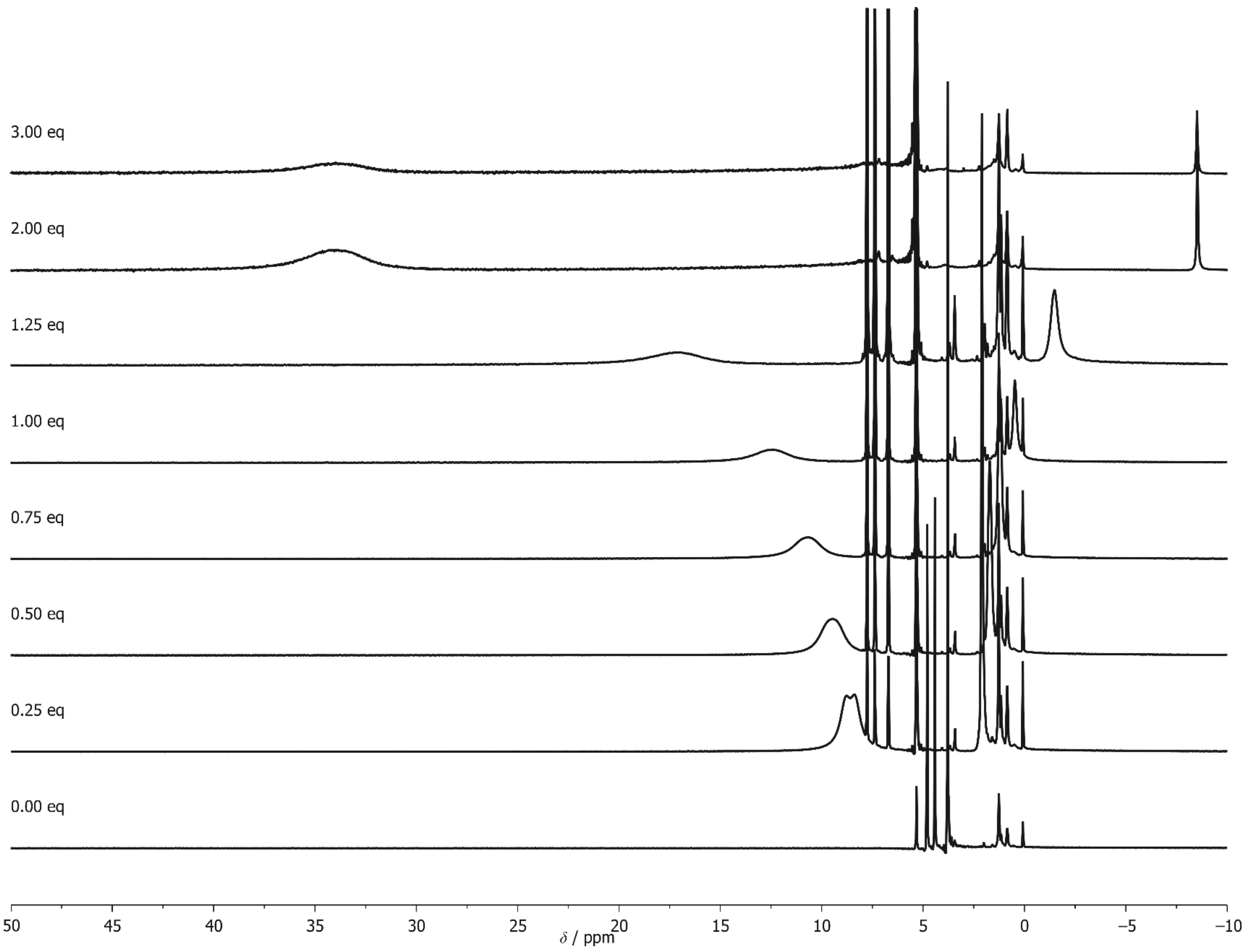

Figure S37: ^{1}H NMR oxidation titration of **2** in CD_2Cl_2 with $[N(2,4\text{-}C_6H_3Br_2)_3]^+$ as oxidant.

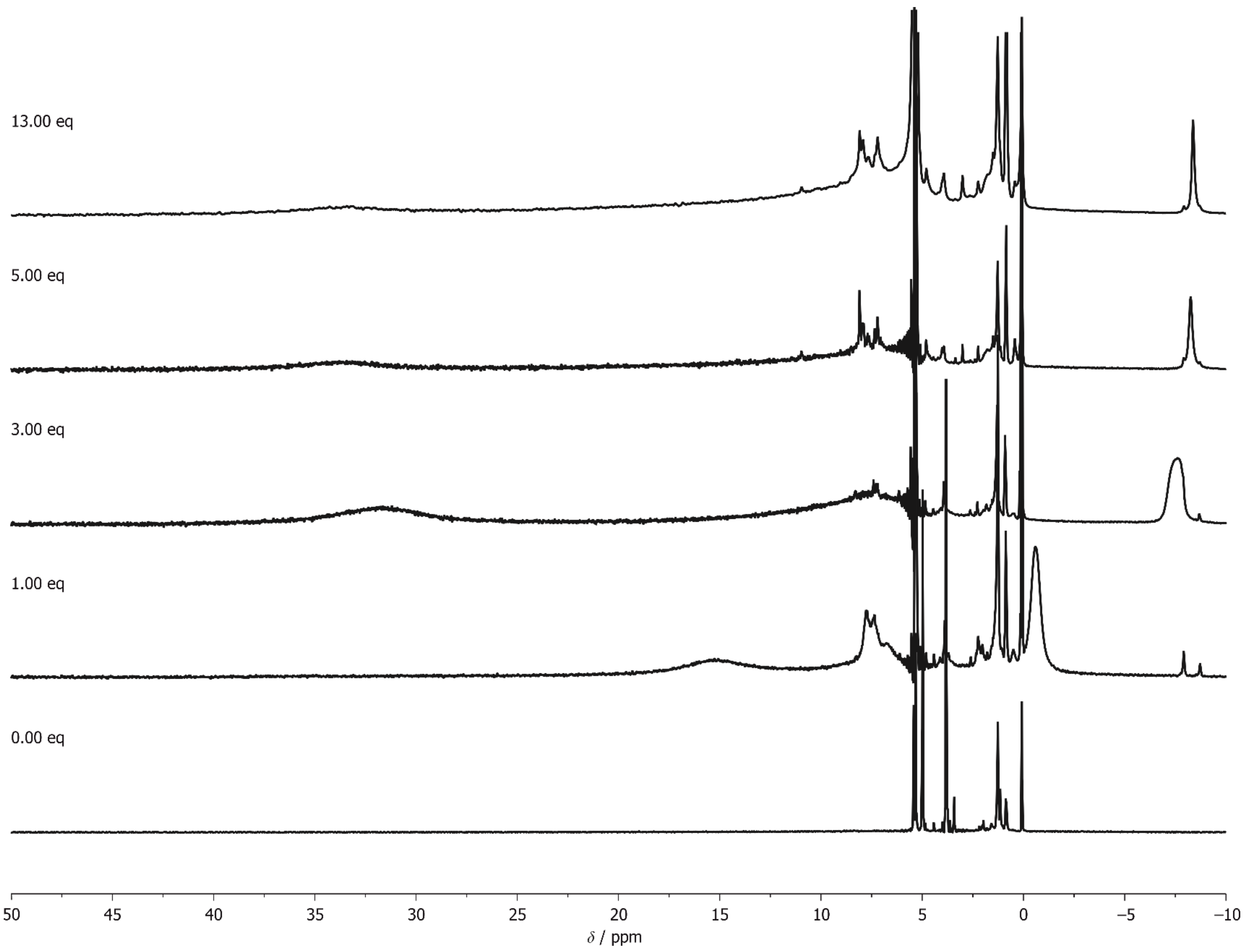

Figure S38: ^{1}H NMR oxidation titration of **4** in CD_2Cl_2 with $[N(2,4\text{-}C_6H_3Br_2)_3]^+$ as oxidant.

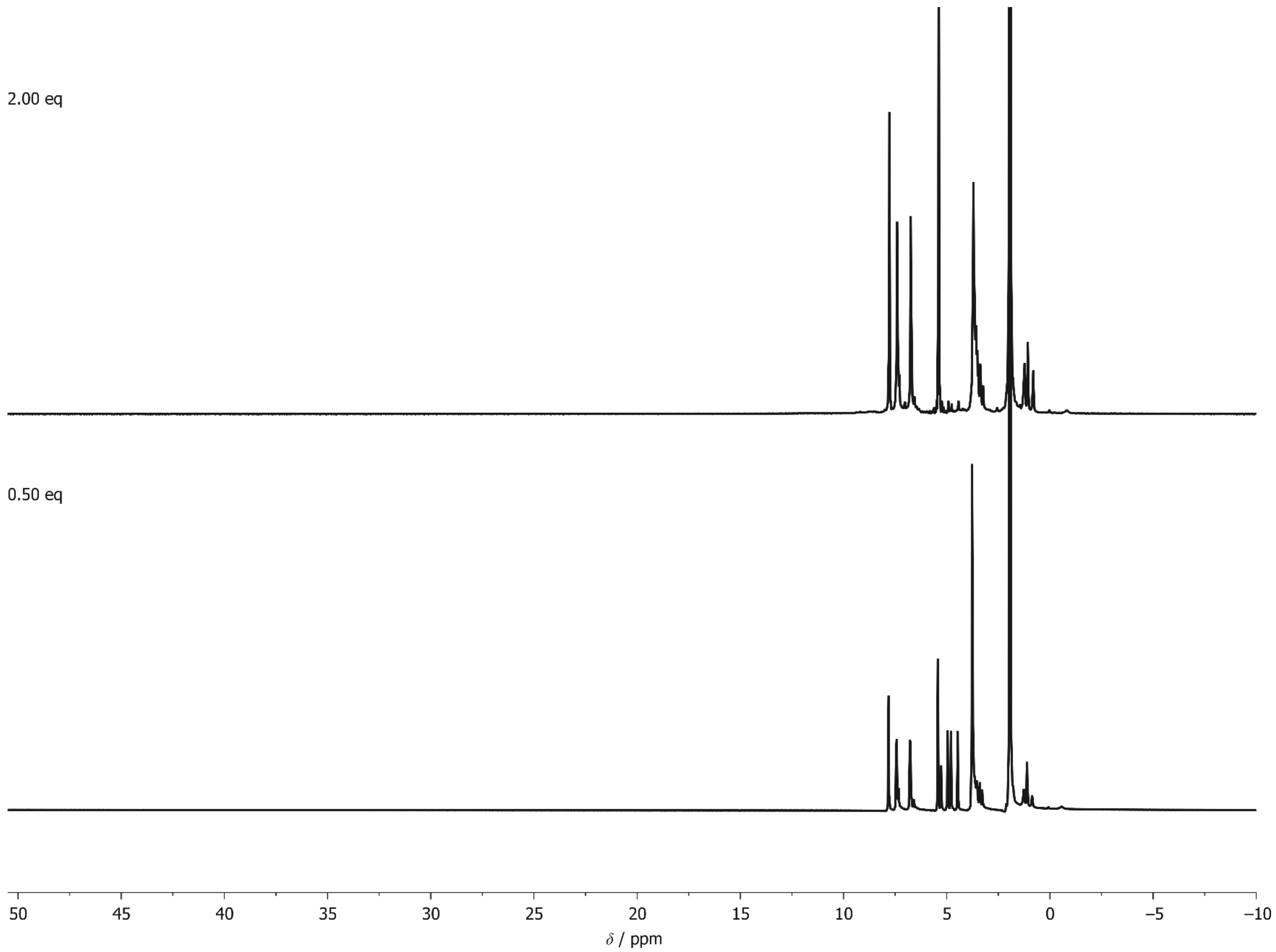

Figure S39: ^{1}H NMR oxidation titration of **3** in CD_3CN with $[N(2,4\text{-}C_6H_3Br_2)_3]^+$ as oxidant.

Cartesian Coordinates of DFT calculated structures

FcH

Fe	-0.494002000	0.000009000	-3.151841000
C	-1.276186000	-1.746125000	-3.947376000
C	0.919806000	-0.363569000	-1.677977000
C	-0.254626000	-1.233517000	-4.797216000
C	-2.297410000	-0.759612000	-3.841429000
C	-0.342764000	-0.070435000	-1.087008000
C	1.309424000	0.759572000	-2.462243000
C	-0.645259000	0.070461000	-5.216672000
C	-1.907841000	0.363540000	-4.625701000
C	-0.733342000	1.233560000	-1.506472000
C	0.288239000	1.746133000	-2.356309000
H	-1.267057000	-2.703627000	-3.451005000
H	1.471475000	-1.283988000	-1.567968000
H	0.663629000	-1.735194000	-5.059115000
H	-3.196435000	-0.841503000	-3.251209000
H	-0.915562000	-0.731252000	-0.455474000
H	2.208445000	0.841416000	-3.052474000
H	-0.072492000	0.731299000	-5.848213000
H	-2.459554000	1.283933000	-4.735716000
H	-1.651583000	1.735266000	-1.244577000
H	0.279150000	2.703633000	-2.852684000

FcH^+

Fe	1.335757000	1.695318000	10.993955000
C	1.083028000	2.490474000	9.056809000
C	2.020982000	3.252137000	9.813820000
H	1.916852000	4.287077000	10.097738000
C	3.112177000	2.392578000	10.142664000
H	3.979570000	2.660611000	10.724346000
C	2.842702000	1.111830000	9.585060000
H	3.456963000	0.231758000	9.690539000
C	1.594931000	1.174024000	8.919122000
H	1.093334000	0.348662000	8.439303000
C	-0.505200000	1.721094000	12.010985000
H	-1.417814000	2.083769000	11.565853000
C	0.466085000	2.511998000	12.691478000
H	0.406353000	3.572873000	12.874102000
C	1.533083000	1.648128000	13.081423000
C	1.210875000	0.333520000	12.647666000
H	1.834954000	-0.539045000	12.756602000
C	-0.041612000	0.379536000	11.988903000
H	-0.531758000	-0.449959000	11.504621000
H	0.137063000	2.844898000	8.679875000
H	2.434454000	1.942484000	13.594494000

1

Fe	-1.670015000	0.023717000	-0.625668000
C	-2.400272000	-1.715480000	-1.452128000
C	-0.691018000	-0.409382000	1.147387000
C	-1.494912000	-1.079962000	-2.363275000
C	-3.474807000	-0.802195000	-1.191538000
C	-1.760933000	0.498212000	1.389909000
C	0.207040000	0.200338000	0.225363000
C	-2.013933000	0.203647000	-2.665615000
C	-3.233929000	0.373938000	-1.947407000
C	-1.523122000	1.672481000	0.617644000
C	-0.306168000	1.489055000	-0.099310000
H	-0.590838000	-1.398289000	1.566195000
H	-0.580380000	-1.510428000	-2.736094000
H	-4.312258000	-0.983001000	-0.538985000
H	-2.614379000	0.315805000	2.023820000
H	1.104208000	-0.244483000	-0.173900000
H	-1.550079000	0.935682000	-3.306991000
H	-3.854949000	1.255308000	-1.957886000
H	-2.167201000	2.535846000	0.562125000
H	0.133998000	2.191834000	-0.788809000
C	-2.201325000	-3.067528000	-0.914593000
O	-1.283998000	-3.798879000	-1.234695000
O	-3.156312000	-3.433868000	-0.040721000
C	-3.038962000	-4.760347000	0.513359000
H	-3.010920000	-5.502985000	-0.283026000
H	-2.137452000	-4.841106000	1.119818000
H	-3.922384000	-4.896815000	1.130608000

1^{+}

Fe	-1.662875000	0.059245000	-0.599781000
C	-2.411617000	-1.712844000	-1.403146000
C	-0.597830000	-0.334570000	1.147342000
C	-1.511493000	-1.086949000	-2.326435000
C	-3.516163000	-0.828250000	-1.204787000
C	-1.641563000	0.576704000	1.473618000
C	0.243195000	0.290944000	0.178734000
C	-2.068404000	0.167222000	-2.686020000
C	-3.299262000	0.321903000	-1.995270000
C	-1.449742000	1.756273000	0.713146000
C	-0.290453000	1.584872000	-0.087667000
H	-0.469919000	-1.325192000	1.553214000
H	-0.583025000	-1.505064000	-2.678366000
H	-4.350538000	-0.995235000	-0.543476000
H	-2.464855000	0.382629000	2.143305000
H	1.117058000	-0.142460000	-0.279302000
H	-1.615983000	0.893300000	-3.342294000

H	-3.934137000	1.193446000	-2.024853000
H	-2.108887000	2.610175000	0.696570000
H	0.094538000	2.296364000	-0.800438000
C	-2.187247000	-3.064485000	-0.825445000
O	-1.155005000	-3.679121000	-0.976656000
O	-3.246862000	-3.512022000	-0.154688000
C	-3.127146000	-4.836157000	0.425611000
H	-2.907962000	-5.562588000	-0.354222000
H	-2.337946000	-4.844302000	1.175317000
H	-4.091231000	-5.036990000	0.882100000

2

Fe	1.322174000	1.659373000	10.977918000
O	-0.864534000	1.808863000	7.700754000
O	-0.644332000	3.860215000	8.594291000
O	3.730745000	0.838353000	13.812879000
O	3.066792000	2.967620000	14.115208000
C	1.048803000	2.296710000	9.025623000
C	1.919591000	3.195269000	9.727368000
H	1.738095000	4.241951000	9.906149000
C	3.053933000	2.455662000	10.145490000
H	3.882771000	2.843413000	10.716719000
C	2.891564000	1.103665000	9.720179000
H	3.575880000	0.293110000	9.912649000
C	1.657793000	1.001902000	9.032801000
H	1.234651000	0.110819000	8.599064000
C	-0.464923000	1.828757000	12.041135000
H	-1.360161000	2.297968000	11.665485000
C	0.584518000	2.486787000	12.729307000
H	0.623400000	3.532539000	12.984520000
C	1.595382000	1.511203000	13.015970000
C	1.153541000	0.254491000	12.492313000
H	1.703777000	-0.670033000	12.546763000
C	-0.115642000	0.453582000	11.895555000
H	-0.705101000	-0.298734000	11.396855000
C	-0.237341000	2.598713000	8.378693000
C	-1.905950000	4.230178000	7.996247000
H	-1.853303000	4.138699000	6.911886000
H	-2.069965000	5.263697000	8.286552000
H	-2.702436000	3.594797000	8.379330000
C	2.895956000	1.710368000	13.673570000
C	4.332696000	3.250109000	14.751312000
H	4.477988000	2.600244000	15.612340000
H	4.275520000	4.288554000	15.063927000
H	5.148382000	3.107811000	14.043774000

2^+

Fe	1.328139000	1.721512000	10.977669000
O	-0.794936000	1.727455000	7.647116000
O	-0.686807000	3.791693000	8.543174000
O	3.664058000	0.738179000	13.850621000
O	3.120277000	2.905574000	14.139231000
C	1.074727000	2.316941000	8.984141000
C	1.897820000	3.251436000	9.693247000
H	1.671028000	4.288842000	9.874551000
C	3.065388000	2.558744000	10.112322000
H	3.873883000	2.976356000	10.691341000
C	2.968654000	1.211751000	9.666413000
H	3.677604000	0.424849000	9.870701000
C	1.749283000	1.059400000	8.974992000
H	1.365045000	0.146149000	8.549650000
C	-0.480857000	1.931984000	12.100992000
H	-1.359816000	2.436950000	11.732913000
C	0.613743000	2.548974000	12.759344000
H	0.701585000	3.593695000	13.007304000
C	1.585421000	1.531117000	13.028663000
C	1.070156000	0.294171000	12.530193000
H	1.577816000	-0.655909000	12.566348000
C	-0.199056000	0.547763000	11.965032000
H	-0.821685000	-0.176469000	11.463726000
C	-0.232961000	2.561001000	8.319162000
C	-1.969025000	4.118343000	7.946053000
H	-1.902942000	4.045470000	6.861852000
H	-2.175381000	5.137635000	8.254979000
H	-2.731468000	3.437947000	8.319462000
C	2.899472000	1.664922000	13.711056000
C	4.388639000	3.130735000	14.808489000
H	4.471235000	2.479549000	15.675890000
H	4.374141000	4.173352000	15.108100000
H	5.207084000	2.936543000	14.117900000

3

Fe	3.974600000	2.976937000	2.307571000
O	0.421427000	2.508593000	3.898118000
O	4.652068000	5.938959000	4.416759000
O	6.522718000	4.738278000	4.777892000
O	1.175771000	0.477902000	3.291823000
O	7.499028000	3.391024000	1.116439000
O	6.944006000	1.301418000	0.492529000
C	3.946816000	1.435279000	3.704119000
H	4.005771000	0.384582000	3.471901000
C	4.509443000	3.612745000	4.200540000
C	5.028216000	2.298065000	3.974037000
H	6.071460000	2.027952000	3.984448000

C	2.739145000	2.203238000	3.781269000
C	4.785774000	4.288599000	0.926896000
H	5.408527000	5.132761000	1.171284000
C	3.087748000	3.554448000	4.078584000
H	2.404697000	4.377325000	4.199603000
C	5.242366000	2.952033000	0.687342000
C	5.343864000	4.792337000	4.494894000
C	1.391180000	1.628361000	3.613636000
C	4.095369000	2.139374000	0.420279000
H	4.109657000	1.082867000	0.211499000
C	6.624376000	2.445422000	0.746872000
C	3.373453000	4.294419000	0.803711000
H	2.731080000	5.148027000	0.948524000
C	2.949695000	2.969711000	0.491346000
H	1.929665000	2.645938000	0.356784000
C	5.395919000	7.154085000	4.649321000
H	4.684619000	7.958546000	4.487920000
H	6.225431000	7.229324000	3.948422000
H	5.778608000	7.176304000	5.668945000
C	8.871268000	2.973356000	1.265549000
H	9.407674000	3.859897000	1.590102000
H	9.262155000	2.609167000	0.316619000
H	8.947253000	2.187918000	2.016027000
C	-0.930774000	2.003213000	3.845828000
H	-1.562149000	2.842804000	4.119934000
H	-1.051557000	1.187285000	4.556613000
H	-1.167158000	1.649731000	2.843410000

3^+

Fe	4.005412000	3.086691000	2.347027000
O	0.507210000	2.322149000	3.867711000
O	4.574474000	5.921323000	4.597468000
O	6.480291000	4.773777000	4.970753000
O	1.376036000	0.322207000	3.292078000
O	7.485777000	3.265537000	0.941381000
O	6.683088000	1.295332000	0.191075000
C	4.069057000	1.423784000	3.734980000
H	4.185269000	0.393866000	3.435350000
C	4.525711000	3.604601000	4.300016000
C	5.107617000	2.328271000	4.024350000
H	6.163472000	2.108916000	4.011465000
C	2.831144000	2.119448000	3.837226000
C	4.880460000	4.429142000	1.028735000
H	5.595863000	5.178205000	1.326045000
C	3.106380000	3.473135000	4.191412000
H	2.378287000	4.250244000	4.353921000
C	5.183768000	3.089472000	0.620870000

C	5.311817000	4.817505000	4.664020000
C	1.501988000	1.477605000	3.624344000
C	3.953416000	2.443455000	0.301556000
H	3.844920000	1.415107000	-0.004085000
C	6.521204000	2.437852000	0.551081000
C	3.469102000	4.589431000	0.963100000
H	2.920879000	5.477082000	1.236087000
C	2.905738000	3.366800000	0.508259000
H	1.853889000	3.156581000	0.389837000
C	5.243950000	7.172342000	4.898651000
H	4.493145000	7.938581000	4.735529000
H	6.090048000	7.309543000	4.229253000
H	5.583920000	7.172228000	5.932717000
C	8.834934000	2.736988000	0.950749000
H	9.458993000	3.561212000	1.280242000
H	9.112598000	2.409666000	-0.048921000
H	8.899730000	1.902801000	1.646682000
C	-0.837190000	1.787464000	3.752856000
H	-1.493065000	2.609956000	4.017966000
H	-0.961413000	0.955811000	4.442941000
H	-1.018709000	1.454116000	2.732657000

4

Fe	4.279457000	4.558302000	1.616690000
O	7.742196000	3.210167000	2.569276000
O	4.484201000	5.115364000	-2.178796000
O	3.967448000	1.772506000	-1.008011000
O	0.798079000	5.992041000	1.691996000
O	6.561988000	5.506490000	-1.402076000
O	1.786856000	6.728382000	3.575735000
O	1.904054000	2.084530000	-0.158903000
O	6.812744000	3.820797000	4.527585000
C	3.120483000	6.268201000	1.660476000
C	3.806185000	2.589287000	1.176405000
C	5.414496000	3.197711000	2.704671000
C	5.377269000	6.284905000	1.213204000
C	4.695022000	5.840397000	0.035940000
C	4.132732000	3.371670000	3.317506000
C	3.110826000	2.135651000	-0.044192000
C	1.857003000	6.362535000	2.420865000
C	3.294987000	5.825669000	0.316488000
C	6.704204000	3.456297000	3.375428000
C	4.412859000	6.547379000	2.209911000
C	5.212179000	2.720040000	1.377654000
C	5.361121000	5.473809000	-1.231273000
C	3.146767000	2.992921000	2.380545000
C	9.059817000	3.377097000	3.138257000

C	5.031168000	4.763686000	-3.468851000
C	-0.477169000	5.969626000	2.369829000
C	3.387101000	1.344533000	-2.259492000
H	4.176146000	4.482616000	-4.075952000
H	5.546480000	5.618794000	-3.903598000
H	5.723810000	3.929221000	-3.372568000
H	6.445220000	6.388804000	1.317696000
H	4.608289000	6.881499000	3.215309000
H	2.513010000	5.529144000	-0.361263000
H	2.079675000	3.022680000	2.529258000
H	3.959754000	3.744881000	4.313451000
H	5.981723000	2.492360000	0.660691000
H	9.188393000	2.708724000	3.987533000
H	9.207352000	4.407970000	3.457575000
H	9.750127000	3.118612000	2.341005000
H	2.782243000	2.142631000	-2.686241000
H	2.770858000	0.459824000	-2.107424000
H	4.231045000	1.115030000	-2.902306000
H	-1.192692000	5.643253000	1.621271000
H	-0.446723000	5.266966000	3.201284000
H	-0.728216000	6.962646000	2.738863000

4^+

Fe	4.278515000	4.556834000	1.615052000
O	7.736028000	3.201008000	2.577236000
O	4.484157000	5.118850000	-2.177847000
O	3.970651000	1.774486000	-1.017176000
O	0.803093000	6.003646000	1.695385000
O	6.563512000	5.505262000	-1.402811000
O	1.795284000	6.734304000	3.579699000
O	1.905548000	2.085852000	-0.172087000
O	6.799832000	3.812106000	4.532091000
C	3.126384000	6.271055000	1.663491000
C	3.804319000	2.588839000	1.168158000
C	5.407751000	3.192270000	2.703438000
C	5.383049000	6.280070000	1.215354000
C	4.698540000	5.841662000	0.037199000
C	4.124079000	3.366186000	3.312122000
C	3.112071000	2.136755000	-0.054810000
C	1.863613000	6.369375000	2.424447000
C	3.298670000	5.831717000	0.318221000
C	6.695258000	3.448252000	3.379375000
C	4.420087000	6.543547000	2.213201000
C	5.209652000	2.717750000	1.374711000
C	5.362728000	5.474904000	-1.230921000
C	3.141018000	2.990628000	2.370792000
C	9.051542000	3.366381000	3.151455000

C	5.029045000	4.766114000	-3.468473000
C	-0.471945000	5.984339000	2.373730000
C	3.392598000	1.344967000	-2.269154000
H	4.172696000	4.488239000	-4.075151000
H	5.546950000	5.619643000	-3.903259000
H	5.719071000	3.929323000	-3.373281000
H	6.451498000	6.379000000	1.319689000
H	4.617111000	6.873954000	3.219519000
H	2.515222000	5.540460000	-0.360077000
H	2.073387000	3.020854000	2.515641000
H	3.948052000	3.737088000	4.308422000
H	5.981680000	2.491016000	0.660111000
H	9.174903000	2.699857000	4.002984000
H	9.200020000	4.397692000	3.468741000
H	9.744690000	3.104353000	2.357845000
H	2.789196000	2.142835000	-2.698369000
H	2.775312000	0.461054000	-2.116873000
H	4.237689000	1.113813000	-2.909865000
H	-1.188807000	5.661263000	1.625037000
H	-0.443479000	5.280411000	3.204210000
H	-0.719695000	6.977640000	2.744241000
